# Enhancement of recombinant adeno-associated virus activity by improved stoichiometry and homogeneity of capsid protein assembly

**DOI:** 10.1016/j.omtm.2023.101142

**Published:** 2023-10-24

**Authors:** Takayuki Onishi, Michika Nonaka, Takahiro Maruno, Yuki Yamaguchi, Mitsuko Fukuhara, Tetsuo Torisu, Masaharu Maeda, Susan Abbatiello, Anisha Haris, Keith Richardson, Kevin Giles, Steve Preece, Noriko Yamano-Adachi, Takeshi Omasa, Susumu Uchiyama

**Affiliations:** 1 Department of Biotechnology, Graduate School of Engineering, Osaka University, 2-1 Yamadaoka, Suita, Osaka 565-0871, Japan; 2 U-Medico Inc, 2-1 Yamadaoka, Suita, Osaka 565-0871, Japan; 3 Osaka Consolidated Laboratory, Manufacturing Technology Association of Biologics, 2-1 Yamadaoka, Suita, Osaka 565-0871, Japan; 4 Waters Corporation, Milford, MA 01757, USA; 5 Waters Corporation (Micromass UK Ltd), Stamford Avenue, Altrincham Road, Wilmslow SK9 4AX, UK

**Keywords:** adeno-associated virus, analytical ultracentrifugation, charge detection-mass spectrometry, transduction efficiency, viral protein stoichiometry

## Abstract

Studies of recombinant adeno-associated virus (rAAV) revealed the mixture of full particles with different densities in rAAV. There are no conclusive results because of the lack of quantitative stoichiometric viral proteins, encapsidated DNA, and particle level analyses. We report the first comprehensive characterization of low- and high-density rAAV serotype 2 particles. Capillary gel electrophoresis showed high-density particles possessing a designed DNA encapsidated in the capsid composed of (VP1 + VP2)/VP3 = 0.27, whereas low-density particles have the same DNA but with a different capsid composition of (VP1 + VP2)/VP3 = 0.31, supported by sedimentation velocity-analytical ultracentrifugation and charge detection-mass spectrometry. *In vitro* analysis demonstrated that the low-density particles had 8.9% higher transduction efficacy than that of the particles before fractionation. Further, based on our recent findings of VP3 clip, we created rAAV2 single amino acid variants of the transcription start methionine of VP3 (M203V) and VP3 clip (M211V). The rAAV2-M203V variant had homogeneous particles with higher (VP1+VP2)/VP3 values (0.35) and demonstrated 24.7% higher transduction efficacy compared with the wild type. This study successfully provided highly functional rAAV by the extensive fractionation from the mixture of rAAV2 full particles or by the single amino acid replacement.

## Graphical abstract

**Figure:**
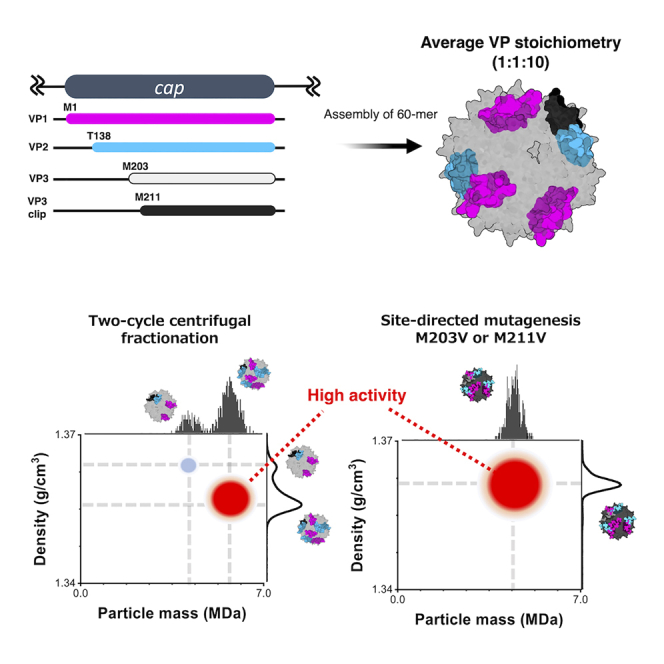

## Teaser

Uchiyama and colleagues discovered protein heterogeneity in rAAV particles and enhanced them through a single amino acid substitution. This is crucial for improving the accuracy and control of VP stoichiometry in gene therapy, where rAAV vectors inherently exhibit variability in VP composition.

## Introduction

Recombinant adeno-associated virus (rAAV) vectors have been used for *in vivo* gene therapy. Several therapeutic products based on rAAV have already been approved for use in human gene therapy.^1^^,^^2^^,^^3^^,^^4^^,^^5^ Adeno-associated virus (AAV) capsid consists of three viral proteins (VPs), namely, VP1, VP2, and VP3, which assemble to form the T = 1 icosahedral capsid composed of 60 VPs. VP1, VP2, and VP3 are present in the AAV capsid at the average molar ratio of 1:1:10,^6^^,^^7^ as determined through gel densitometry. Meanwhile, we have also recently reported the existence of VP3 clip in some serotypes of rAAV, which is eight amino acid residues shorter than VP3.^8^^,^^9^ The icosahedral capsid is assembled by the VP3 common region, whereas the N-terminal extensions of VP1 and VP2 play crucial roles in endosomal trafficking^10^^,^^11^ and escape,^12^^,^^13^ nuclear localization, and genome release. Therefore, AAV particles with high VP1 and VP2 stoichiometry are required to develop AAVs that can be safely used in gene therapy because high doses of low-potency AAVs can cause adverse immune reactions, making their application in gene therapy problematic.

The VP subunits of the AAV capsid are randomly incorporated, and mass spectrometry and mass spectral simulation have suggested that they exhibit particle heterogeneity.^14^^,^^15^ Cesium chloride (CsCl) isopycnic density centrifugation could be used for the fractionation of heterogeneous particles. Wang et al. reported that rAAV8 preparations showed low-density and high-density full particles in CsCl isopycnic centrifugation.^16^ The two types of particles contained similar packaged DNA, but the high-density particles had a higher DNA:protein ratio than the low-density particles. In addition, the high-density particles have been found to exhibit lower VP1 and VP2 ratio than the low-density particles through gel densitometry. Previous studies have reported the existence of low- and high-density particles that represent heterosis of AAVs in the CsCl isopycnic gradient, by focusing not only on capsid protein^7^^,^^16^^,^^17^ but also on DNA length as the cause of heterosis.^18^^,^^19^ On the other hand, in the wild-type (WT) AAVs, contradictory findings have been reported, i.e., that both types of particles were reported to have the same DNA:protein ratio and density in sucrose metrizamide gradients.^19^ As described earlier, the VP ratio affects transduction efficacy.^12^^,^^20^^,^^21^^,^^22^^,^^23^^,^^24^ Therefore, accurate quantification and control of the stoichiometry of VPs is becoming increasingly important. Moreover, in the manufacturing of rAAV, the focus is currently on the scaling up production.^25^^,^^26^^,^^27^ In the future, it will be necessary to resolve heterogeneity, which receives little attention from a manufacturing perspective.

Here, we comprehensively characterized low- and high-density particles that were fractionated using a high-resolution fractionation method. Particle heterogeneity given by particles of two different densities could be the packaged DNA or the VP ratio or post-translational modifications of VPs. Recently, highly sensitive and quantitative protein and DNA analysis methods based on capillary gel electrophoresis (CGE) have been developed. Moreover, we reported a quantitative evaluation method for VP stoichiometry using CGE and liquid chromatography-mass spectrometry (LC-MS).^9^ This method allows accurate and reliable estimation of the stoichiometry of VPs in rAAV particles. We have used this quantitation method to evaluate the fractions of high- and low-density particles during rAAV2 production. Typically, one cycle of CsCl isopycnic ultracentrifugation is used for the fractionation; however, the two types of particles remain mixed together even after the fractionation because of the marginal difference in their density. We thus utilized two-cycle purification through ultracentrifugation with separation conditions optimized according to CsCl density gradient simulation. Using this approach, comprehensive assessments of the physicochemical properties and *in vitro* potency of the rAAV particles in the well-separated fractions at the particle level were possible.

Meanwhile, we successfully obtained rAAV variants with increased VP1 and VP2 stoichiometry compared with that of WT rAAV by producing amino acid substitutions at positions M203 and M211, the translation initiation sites of VP3 and VP3 clip, which were previously considered to be associated with a lack of particle formation. Furthermore, the distribution of rAAV particles became highly homogeneous. In this study, we successfully obtained functional rAAV particles with high potency and less protein heterogeneity through two cycles of CsCl ultracentrifugation or amino acid substitution. Based on these findings, this study proposes a potentially novel approach to capsid-based rAAV development.

## Results

### Comprehensive physicochemical characterization of high- and low-density particles

**Figure 1:**
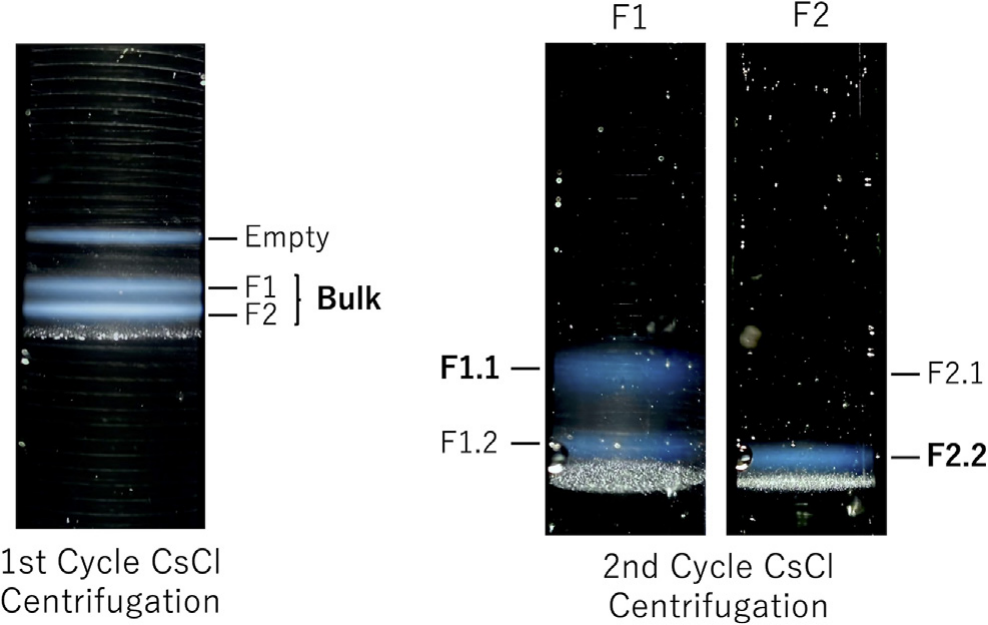
Illustration of two-cycle CsCl isopycnic density gradient ultracentrifugation High- and low-density rAAV2 particles were separated by performing two cycles of CsCl isopycnic gradient ultracentrifugation after affinity chromatography purification. Purified rAAV2-CMV-EGFP vectors were developed using a suspended HEK293T production system. Two full particle bands were observed, denoted as F1 and F2 (left). In both fractions, considerable mixing of the two types of particles was observed after the first cycle; thus, a second cycle of CsCl ultracentrifugation (right) was conducted for F1 and F2, where the two sets of particles with different densities were observed in both fractions, leading to further fractionation into F1.1, F1.2, F2.1, and F2.2. In another preparation, the F1 and F2 sample fractionated in a mixture denoted as “Bulk”.

**Figure 2:**
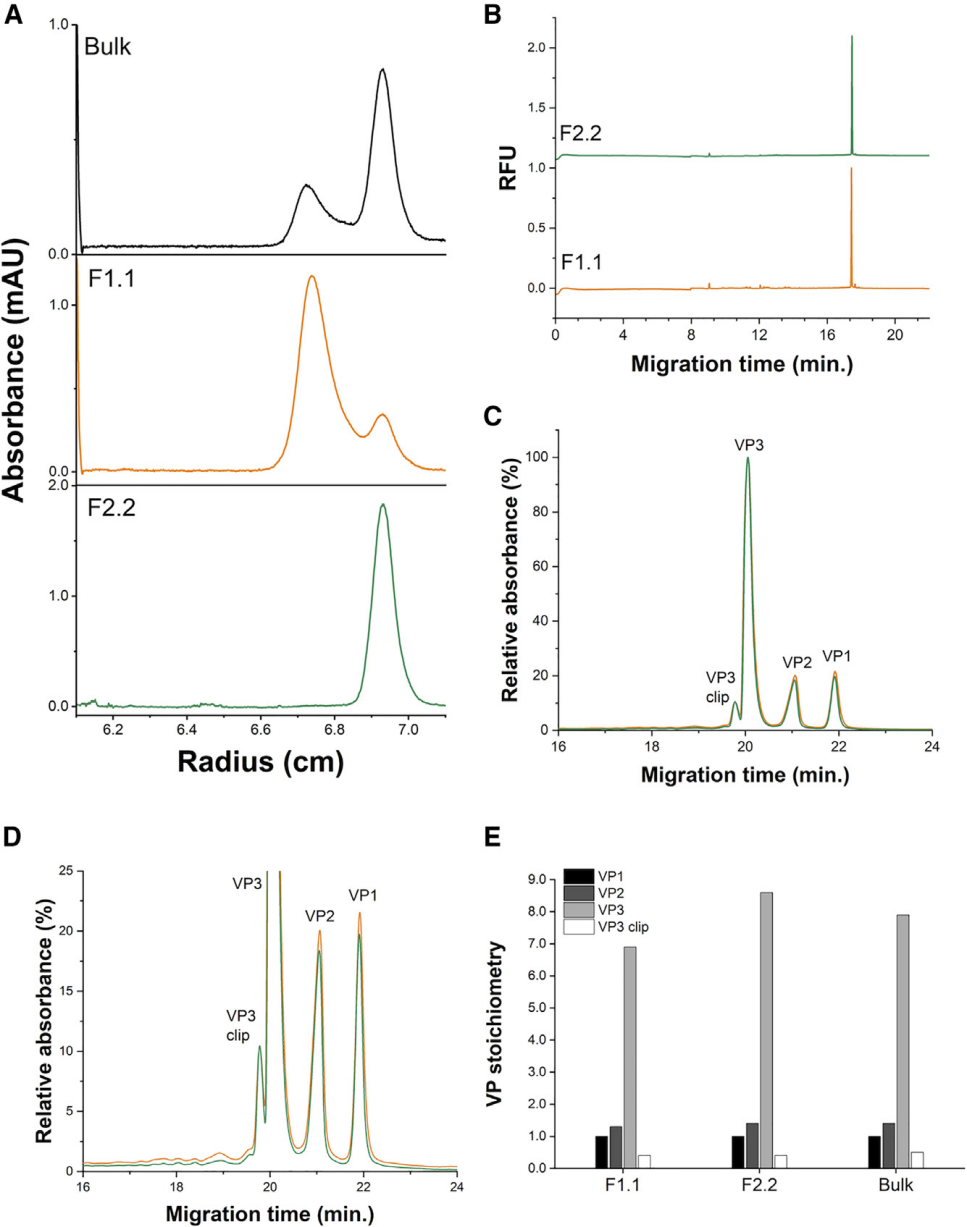
Comprehensive physicochemical characterization of high- and low-density particles (A) CsCl density-gradient-analytical ultracentrifugation (CsCl-DG-AUC) profile of Bulk, F1.1 and F2.2 fractions. (B) Capillary gel electrophoresis (CGE) electropherograms of encapsidated DNA in F1.1 and F2.2 particles. (C) CGE electropherogram of F1.1 and F2.2 particles for capsid viral protein (VP) components. (D) Magnified image of electropherogram shown in (C). (E) VP stoichiometry of Bulk, F1.1, and F2.2 fractions. To determine the VP molar stoichiometry, areas of the CGE peaks detected at 214 nm were divided by the molar extinction efficient of VPs at 214 nm, reflecting the UV absorbance of peptide bonds and amino acids under denatured conditions.

During rAAV production, it would be expected that both low- and high-density full particles would be observed.^16^ Figure 1 illustrates two bands for both particles in CsCl ultracentrifugation, forming two fractions due to the piston fractionator with an online monitoring apparatus, denoted as F1 and F2. In both fractions, significant mixing of the two types of particles was observed; thus, a second cycle of CsCl ultracentrifugation was conducted for F1 and F2, where two sets of particles with different densities were found in both fractions, leading to further fractionation into F1.1, F1.2, F2.1, and F2.2. Based on the results of SDS-PAGE and absorbance measurements, which showed an absence of protein and DNA (data not shown), we concluded that the white layer was precipitated CsCl. The purified low- and high-density full particles were subjected to comprehensive characterization. CsCl density gradient-analytical ultracentrifugation (CsCl-DG-AUC) analysis showed that the two full particles had estimated buoyant densities of 1.352 g/cm^3^ for F1.1 and 1.361 g/cm^3^ for F2.2, with Ab260/280 ratios of 1.277 for F1.1 and 1.280 for F2.2. After the second fractionation, F1.1 still contained a small fraction of high-density full particles; however, low-density full particles could not be detected in F2.2 through CsCl-DG-AUC (Figure 2A). CGE was then performed to analyze the packaged DNA and VP components of F1.1 and F2.2. CGE enables greater separation and sensitivity than those by conventional SDS-PAGE and agarose gel electrophoresis.^28^^,^^29^ CGE for single-stranded DNA (ssDNA) showed that the length of the contained transgene DNA was the same between the high- and low-density particles, indicating no contribution of the packaged DNA genome to the difference in density between these particles (Figure 2B). Electropherograms from CGE for VP proteins with detection at 214 nm and normalized intensity with respect to the VP3 peak are shown in Figures 2C and 2D. All the structural capsid proteins were present in both F1.1 and F2.2 particles. In addition to VP1, VP2, and VP3 peaks, we also detected VP3 clip generated by ribosomal leaky scanning of the first initiation codon of VP3. The analysis suggested that the F1.1 capsids contained a higher ratio of VP1 and VP2 to VP3 than that of the F2.2 capsids. The VP stoichiometry for these particles was determined by calculating the peak area, using the molar absorption coefficient at 214 nm estimated from the amino acid sequence of each VP protein^9^^,^^30^ (Figures 2C and 2D). The ratio of VP1:VP2:VP3:VP3_clip_ was 1.0:1.3:6.9:0.4 for FP1.1 and 1.0:1.4:8.6:0.4 for FP2.2, respectively (Figure 2E), which correspond to (VP1 + VP2)/VP3_total_ of 0.32 for FP1.1 and 0.25 for FP2.2, where VP3_total_ is the sum of VP3 and VP3 clip. There was clear difference in VP stoichiometry between the two full particles, which was highly likely to generate the different buoyant densities for the two full particles.

### F1.1 particles have higher mass than that of F2.2 particles

**Figure 3:**
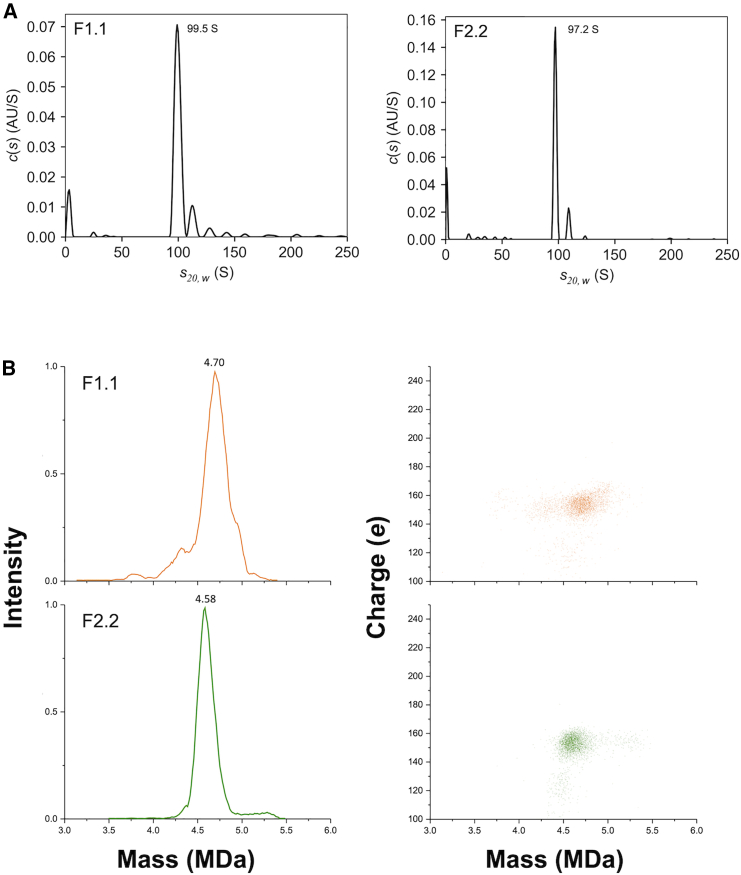
Particle mass analysis of F1.1 and F2.2 particles (A) Sedimentation profiles of F1.1 and F2.2. (B) CDMS mass histogram (right) and charge versus mass scatterplot (left) for F1.1 and F2.2 particles.

As F2.2 particles contained low VP1 and VP2 stoichiometry, it is reasonable to suspect that the molecular weight of the high-density particles could differ from that of the low-density particles. Therefore, band sedimentation-analytical ultracentrifugation (BS-AUC) and charge detection-mass spectrometry (CDMS) measurements were conducted for the particles in F1.1 and F2.2. BS-AUC enables evaluation of the size distribution in solution with only 1/25 of the sample volume compared with that required for sedimentation velocity-analytical ultracentrifugation.^31^ The sedimentation profile showed that F1.1 and F2.2 included no empty particles, and the sedimentation coefficients of F1.1 and F2.2 particles were 99.5 S and 97.2 S, respectively (Figure 3A). According to Equations S1 and S2, the increase in the sedimentation coefficient of these F1.1 particles indicates that the average molecular weight of the F1.1 particles was increased compared with that of the F2.2 particles. Additionally, CDMS measurements revealed the single mass of F1.1 and F2.2 particles as 4.70 MDa and 4.58 MDa, respectively (Figure 3B). Considering the possible influence of counterions, salt adducts and trapped solvent as suggested by Jarrold^32^ and that CGE provided average VP stoichiometry, a reasonably good correspondence was found between experimental and theoretical values calculated by converting the VP ratio from CGE to 60-mer and multiplying by the mass of each VP (Table S1). A low charge population was also presented in charge versus mass scatterplot of CDMS (Figure 3B). According to the CDMS study, the ejection of encapsidated DNA from AAV will result in the higher charged state while compact, near spherical geometries of AAV will lead to the lower charged state.^33^ The relative comparison of the molecular mass obtained from both BS-AUC and CDMS showed higher value in F1.1 than F2.2. Because the DNA content was the same, the increase in mass could be attributed to the higher VP1 and VP2 ratios in the 60-mers. This in turn supported the assertion that the VP1 and VP2 stoichiometry in the F1.1 particles is higher than that in the F2.2 particles.

### *In vitro* transduction efficacy of F1.1 and F2.2

**Figure 4:**
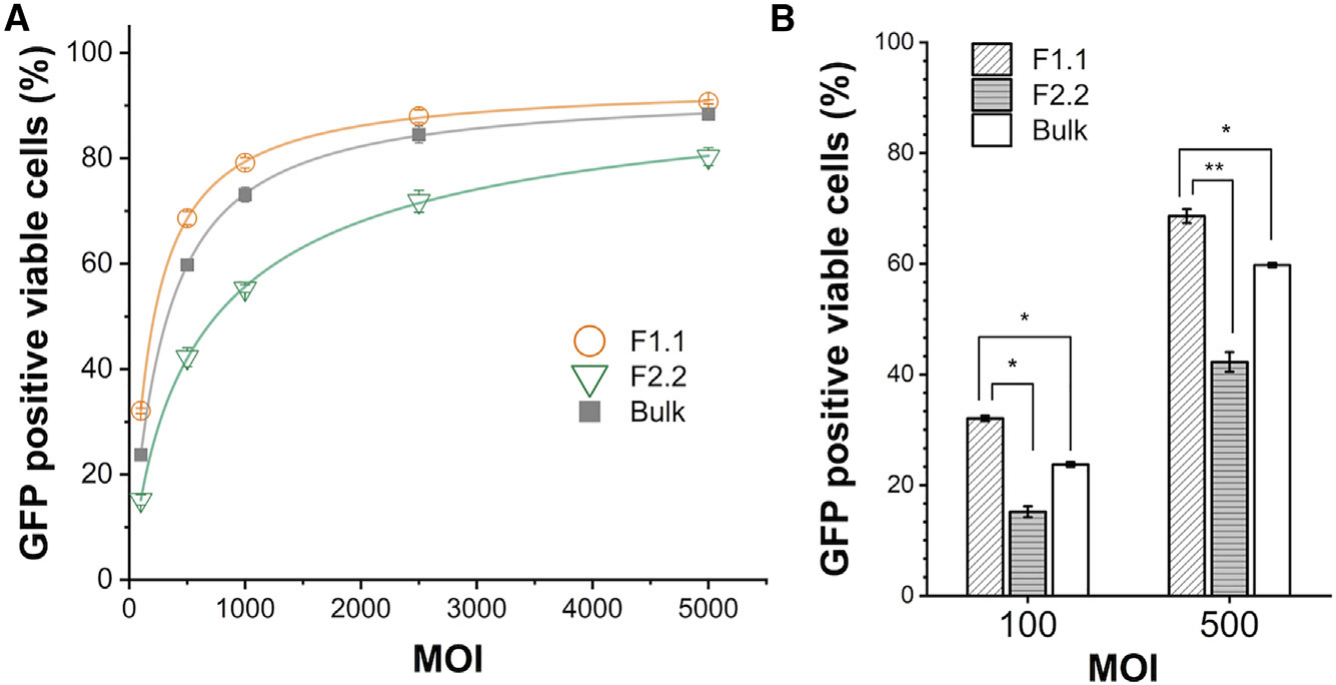
*In vitro* transduction efficacy of F1.1 and F2.2 particles in HelaRC32 cells GFP reporter gene assay results are presented as the mean from triplicate wells in one representative experiment out of two independent runs. Error bars represent ± SD of the three independent wells. (A) GFP-positive viable cells were measured using a fluor cytometer at five MOI points (MOI 1 × 10^2^, 2.5 × 10^3^, 1 × 10^3^, 2.5 × 10^3^, and 5 × 10^3^). (B) *In vitro* transduction efficacy of selected MOIs (1× 10^2^ and 5 × 10^2^).

We hypothesized that the virus could require fewer particles for infectivity if the stoichiometry is biased toward VP1, which is directly related to the presence of the phospholipase A2 (PLA2) domain on the VP1 unique (VP1u) sequence.^11^^,^^23^ Furthermore, the VP1/VP2 common region has a nuclear localization sequences (NLSs).^10^ Therefore, these N-terminal extensions of VP1 and VP2 play crucial roles in endosomal trafficking and escape, nuclear localization, and genomic release, and the total amount of VP1 and VP2 significantly affects transduction efficiency. The biological activity was evaluated using the expression level of a transgene, GFP, encoded in the rAAV2 genome. The proportion of GFP-positive viable cells was evaluated by flow cytometry at five different multiplicity of infection (MOI) levels. The infectivity of F1.1 was up to 8.9% (MOI 5 × 10^2^) higher and F2.2 up to 17.8% (MOI 1 × 10^3^) lower compared to Bulk (Figures 4A and 4B). These results confirmed that VP1 and VP2 stoichiometry plays a crucial role in infectivity *in vitro*, consistent with previous studies of Bosma et al., who reported an interplay between VP1 and VP2 stoichiometry and infectivity *in vitro*.^24^

### Change in the translation initiation site of VP3 and VP3 clip alters the stoichiometry of VP1:VP2:VP3_total_

**Figure 5:**
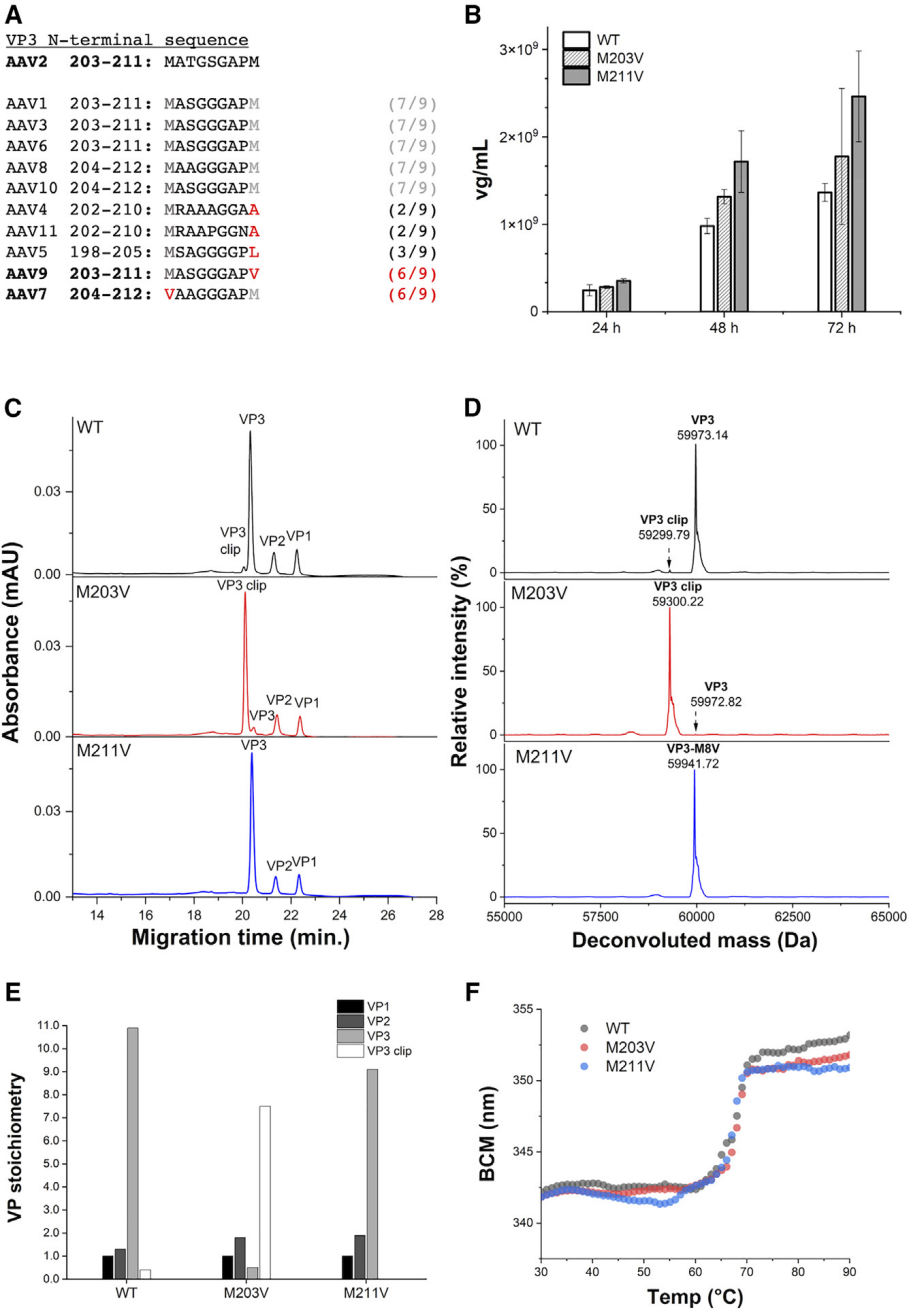
Characterization of rAAV2-M203V and -M211V (A) Amino sequence alignment of the VP3 N-terminal region of AAV1-11 and the number of matches with the AAV2 sequence. AAV7 and AAV9 had a the highly conserved sequence with AAV2 for the VP3 N-terminal region. (B) Productivity comparison between rAAV2 wild-type (WT) and rAAV2 variants at 24, 48, and 72 h post-transfection. Productivity assay results are presented as the means of three independent production batches. Error bars represent ± SD of titer in three independent production batches. (C) CGE electropherograms of rAAV2-WT and rAAV2 variants. (D) Deconvoluted mass values of rAAV2-WT and rAAV2 variants for VP3 and VP3 clip. (E) VP stoichiometry of rAAV2-WT and rAAV2 variants. To determine the VP molar stoichiometry, areas of the CGE peaks detected at 214 nm were divided by the molar extinction efficient of VPs at 214 nm, reflecting the UV absorbance of peptide bonds and amino acids under denatured conditions. (F) Thermal unfolding plot of rAAV2-WT and rAAV2 variants.

rAAV variants by site-directed mutagenesis at the translation initiation sites of VP3 and VP3 clip were created to determine whether these rAAV variants could alter the level of VP3 incorporation into particles. This was based on the report by Bosma et al. that showed that the level of VP3 incorporation affects the incorporation of VP1 and VP2.^24^ However, mutations at the translation initiation site of VP3 and VP3 clip, located at positions M203 and M211, have been reported to prevent particle recovery during production, suggesting that these residues play a crucial role in the assembly and/or stability of the particles of rAAV2.^34^ We then focused on AAV7 and AAV9, which have highly homologous N-terminal sequences of VP3 among the serotypes and conserved sequences, other than ATG at the start codon (Figure 5A). Through serotype sequence alignment, we noticed that, in AAV7, position M203 is CTG (valine), whereas in AAV9, position M211 is CTG (valine). We thus created constructs with CTG substitution at the ATG translation start site for each (rAAV2-M203V and rAAV2-M211V). Interestingly, these rAAV variants exhibited productivity comparable to that of WT rAAV2 (Figure 5B), and they showed less variation in productivity across several batches (data not shown). Meanwhile, DNA packaging ratio of rAAV variants were no significant difference compared with that of WT (Figure S1).

CGE and LC-MS were performed to evaluate the VP components of both rAAV2-M203V and rAAV2-M211V. rAAV2-M203V particles significantly incorporated VP3 clip, whereas rAAV2-M211V particles incorporated full-length VP3 besides VP1 and VP2 (Figure 5C). The deconvoluted mass from LC-MS analysis that provided the mass of each VP in these rAAV variants corresponded to the theoretical values by <25 ppm (Figures 5D and S2; Table S2). VP stoichiometries of rAAV2-M203V and rAAV2-M211V were calculated from the molar absorption coefficient at 214 nm of each denatured VP and the peak area of CGE (Figure 5E). VP1 and VP2 stoichiometry was indicating a predominant trend in rAAV2-M203V and rAAV2-M211V such that (VP1 + VP2)/VP3_total_ = 0.35 and 0.32, respectively (Figure S3).

**Figure 6:**
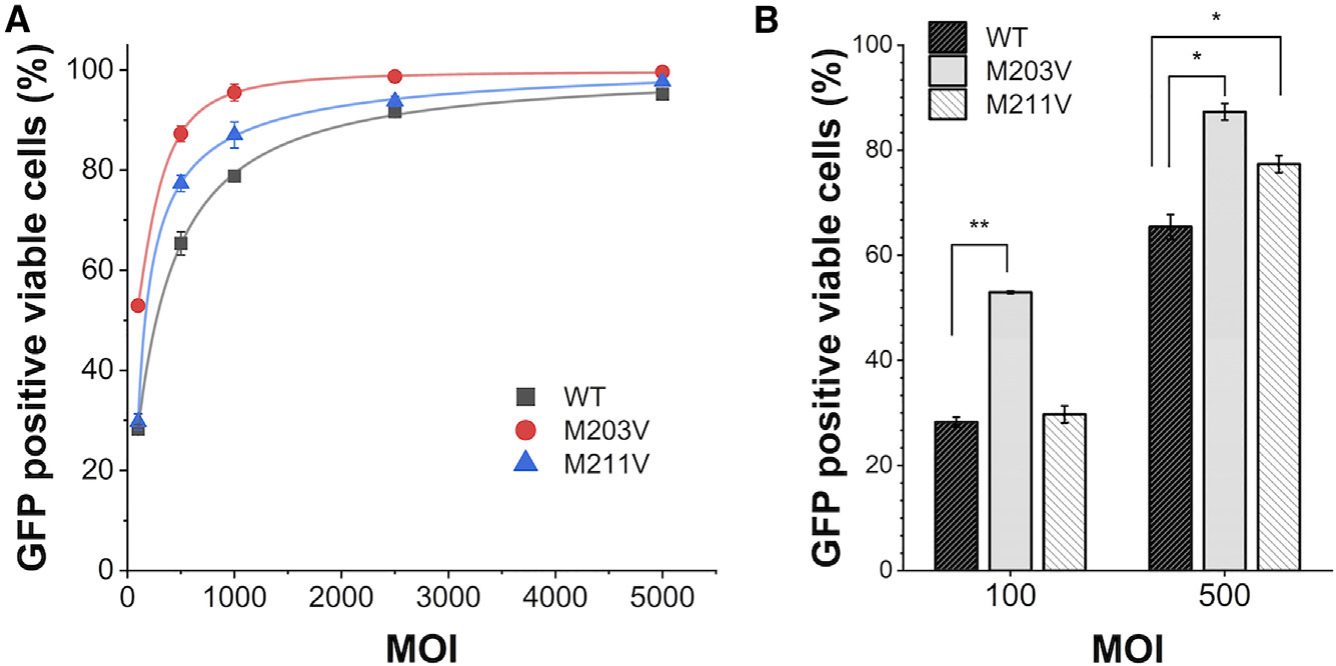
*In vitro* transduction efficacy of rAAV2-M203V and -M211V in HelaRC32 cells GFP reporter gene assay results are presented as the mean from triplicate wells in one representative experiment out of two independent runs. Error bars represent ± SD of the three independent wells. (A) GFP-positive viable cells were measured using a fluor cytometer at five MOI points (MOI 1 × 10^2^, 5 × 10^3^, 1 × 10^3^, 2.5 × 10^3^, and 5 × 10^3^). (B) *In vitro* transduction efficacy of selected MOIs (1 × 10^2^ and 5 × 10^2^).

The denaturation temperatures (*T*_m_) of these rAAV variants were assessed by differential scanning fluorimetry (DSF) to be 67.1°C for WT rAAV2, 68.2°C for rAAV2-M203V, and 67.7°C for rAAV2-M211V, indicating that these mutations had negligible impact on the conformational stability of the particles (Figure 5F). rAAV2-M203V and rAAV2-M211V showed 21.9% (MOI 5 × 10^2^) to 24.7% (MOI 1 × 10^2^) and 8.2% (MOI 2.5 × 10^3^) to 11.9% (MOI 1 × 10^3^) higher *in vitro* transduction effects on HeLaRC32 cells compared with WT rAAV, respectively (Figure 6). These results were consistent with high VP1 and VP2 stoichiometry showing high transduction efficiency and replicated with different genes of interest (Figure S4).

### rAAV2-M203V and rAAV2-M211V possess relatively homogeneous capsid stoichiometry

**Figure 7:**
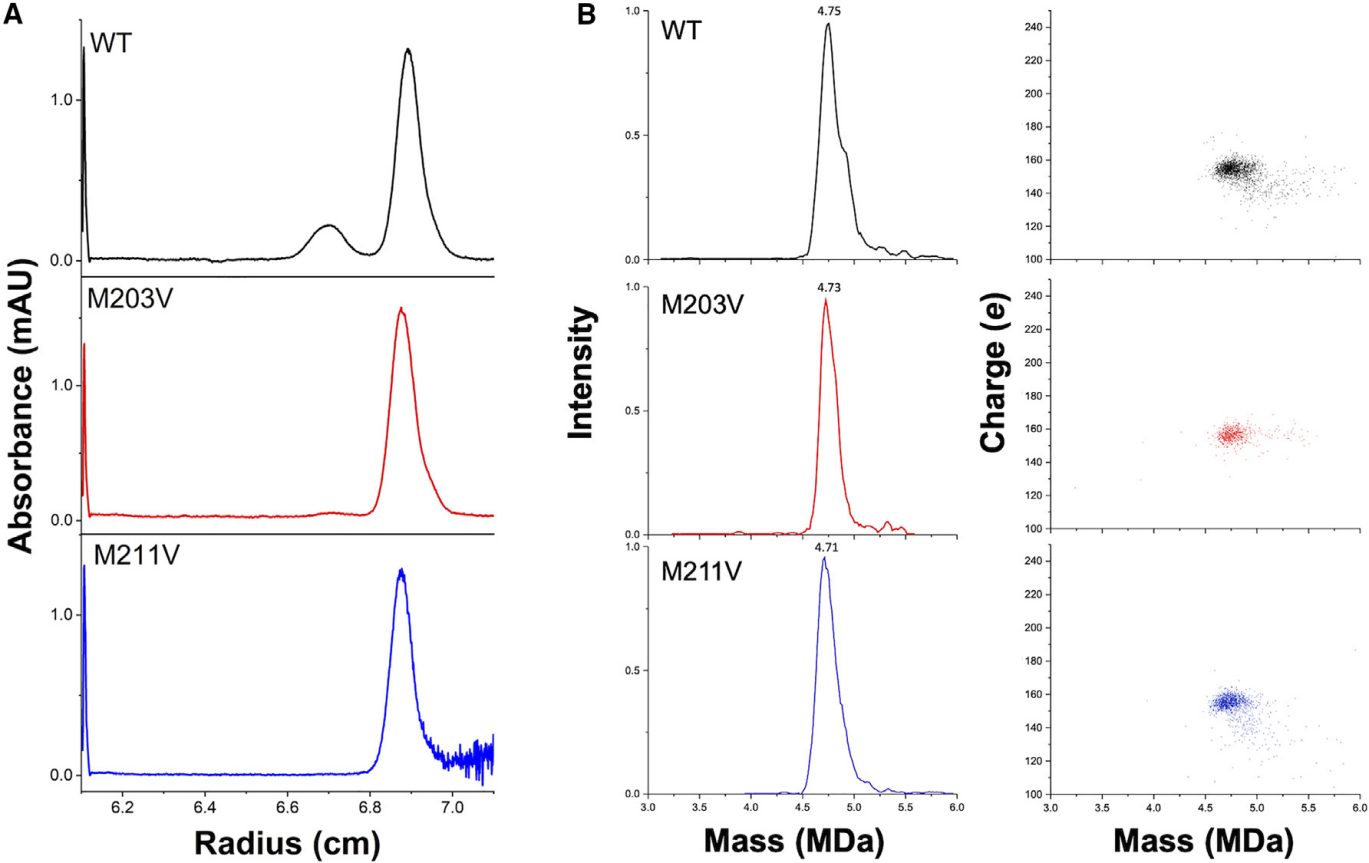
Particle homogeneity of rAAV2-M203V and -M211V (A) CsCl-DG-AUC profile of rAAV2-WT, -M203V, and -M211V for bulk state. (B) CDMS mass histogram (right) and charge versus mass scatterplot (left) of rAAV2-WT, -M203V, and -M211V for bulk state.

We noticed that the particle heterogeneity of these rAAV variants was altered during rAAV purification. CsCl-DG-AUC was performed to measure the particle density distribution for each variant in a bulk state. As a result, the distribution of the particles into two different densities in WT rAAV converged to a single density in rAAV2 variants (Figures 7A and S5). The estimated densities from CsCl-DG-AUC of both rAAV2-M203V and rAAV2-M211V were 1.359 g/cm^3^.

The theoretical molecular weights of rAAV2-WT, -M203V, and -M211V calculated using the VP stoichiometry derived from CGE were 4.54, 4.56, and 4.57 MDa, respectively. Particle measurements with CDMS for the bulk samples of rAAV2-WT, -M203V, and -M211V gave molecular weights of 4.75, 4.73, and 4.71 MDa, respectively (Figure 7B), and these values were in close agreement with the theoretical values. Notably, compared with bulk WT rAAV2, the convergence of molecular mass to homogeneity was confirmed for the two rAAV2 variants by the full width at half maximum (FWHM) values of mass histogram. The FWHM values for rAAV2-WT, -M203V, and -M211V were 0.18 MDa, 0.13 MDa, and 0.16 MDa, with the rAAV variant having smaller values than WT. The sharpness of the peak in CDMS mass histogram was in the following order: rAAV2-WT> -M211V > -M203V. These results revealed that the two rAAV2 variants have reduced particle heterogeneity.

### Total VP1 and VP2 stoichiometry affects *in vitro* transduction efficacy

**Figure 8:**
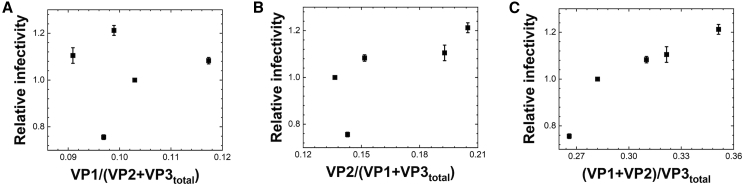
Correlation between VP stoichiometry and transduction efficacy (A–C) Plot of VP molar stoichiometry and transduction efficiency of Bulk, F1.1, F2.2, rAAV2-M203V, and -M211V. Relative potencies indicate GFP-positive viable cell counts at MOI 1 × 10^2^, all normalized to the bulk state WT value of 1. Error bars represent ± SD for three independent wells in the GFP reporter gene assay. (A) Correlation between VP1 stoichiometry and relative potency. (B) Correlation between VP2 stoichiometry and relative potency. (C) Correlation between the total stoichiometry of VP1 and VP2 and relative potency.

Based on GFP reporter gene expression results, we normalized the gene transduction efficiencies of F1.1, F2.2, rAAV2-M203V, and rAAV2-M211V using the efficiency of gene transduction of bulk rAAV-WT as measured by flow cytometry. Further, the correlation among the relative VP1 stoichiometry (VP1/[VP2 + VP3_total_]), VP2 stoichiometry (VP2/[VP1 + VP3_total_]), VP1 and VP2 stoichiometry ([VP1 + VP2]/VP3_total_) and the normalized transduction efficiency based on the VP stoichiometry calculated from the area ratio of the CGE and the molar absorption coefficient was depicted (Figure 8). The results showed that there was no good correlation between “VP1 only” and “VP2 only,” indicating good correlation between the VP1 and VP2 stoichiometry and relative transduction efficiency. This was consistent with previous reports that showed the correlation based on the quantitation by gel densitometry, while our current study revealed a clearer numerical relationship about the correlation. This is directly related to the presence of a phospholipase A2 domain on the VP1u sequence and nuclear transfer signals on the VP1 and VP2 sequences. Naturally, compared with those of F1.1, rAAV2-M203V, and -M211V, the significantly lower transduction efficacy at MOI 1 × 10^3^ of F2.2, which contains only high-density rAAV particles, could be explained by the lower VP1 and VP2 stoichiometry.

## Discussion

In this study, we demonstrated the two different distributions of the density of full particles observed in rAAV in the CsCl density gradient originated from the heterogeneity of VP subunits and not from the size difference of the viral genome. The (VP1 + VP2)/VP3_total_ values were 0.31 for F1.1 and 0.27 for F2.2, corresponding to an increase of 1.8% and a decrease of 2.8% compared with Bulk ([VP1 + VP2]/VP3_total_ value, 0.28), respectively. These quantitative relationships could be clarified by the accurate CGE method that we recently developed by combining CGE and mass spectrometry.^9^ The BS-AUC and CDMS results supported the higher VP1 + VP2 number in F1.1 than that in Bulk and F2.2. The estimation of the VP1, VP2, and VP3 combinations in 60-mer based on particle mass determined by CDMS suggested that VP1 and VP2 stoichiometry was obviously higher for the F1.1 particles than for the F2.2 particles (Figure S6). Correspondingly, F1.1 particles purified through two cycles of CsCl ultracentrifugation showed high transduction efficacy *in vitro*, as evidenced by the flow cytometry analysis of GFP reporter gene expression in HeLaRC32 cells and transduction efficiency that showed higher transgene expression for F1.1 than Bulk (up to 8.9%). However, considering the reason for the different densities of AAV in CsCl isopycnic density gradient centrifugation was unclear from the contradictory reports published to date, no definitive conclusions about the relationship between AAV density and potency could be drawn.^7^^,^^16^^,^^17^^,^^18^^,^^19^^,^^35^ As described above, an accurate and reliable method for quantifying the VP stoichiometry was recently developed. For example, SDS-PAGE with a dye staining approach provides less accurate quantitation results because of the dependence of the band intensity on the amino acid composition. In the development of rAAV vector, the VP stoichiometry has been recognized as a critical quality attribute and quality control as a specification for rAAV products as a biopharmaceutical. Meanwhile, how VP ratio of rAAV produced by HEK cells has an impact on the transduction efficacy was unknown so far.

Low- and high-density particles have also been previously observed in WT AAVs (AAV1,^17^ AAV3,^7^ AAV4,^18^ and avian AAV^36^) produced using adenoviruses as helpers and other parvoviruses (H-1 parvovirus^37^ and mouse minute virus [MVM]^38^) preparations. High-density particles of WT AAV2 were reported to have a higher DNA:protein ratio than low-density particles.^7^ However, other studies reported that both types of particles have the same DNA:protein ratio and sedimentation velocity.^19^ Furthermore, the ratio of infectious units to physical particles has been reported to be 16- to 300-fold higher for low-density particles of AAV2 than that for high-density particles.^19^ Conversely, no significant difference was observed in the infectivity between low-density and high-density particles of AAV4, MVM, and H1 parvovirus,^18^^,^^37^^,^^38^ although some studies attributed that the difference in density originated from the difference in the DNA contents of such particles. Meanwhile, the rAAV study concluded that the difference in density originated from the difference in the VP ratio of such particles. This discrepancy regarding the properties of high- and low-density particles of AAV could be attributed to differences in sample preparation method, as some studies used single CsCl ultracentrifugation for fractionation. Especially in the case of the same encapsidated DNA, and density difference derived from VP stoichiometry, as in this case, a single CsCl ultracentrifugation is insufficient to separate the two types of particles into fully distinct fractions, since the difference in estimated density between the two types of particles is marginal (∼0.009 g/cm^3^). In fact, F1.1, which was prepared by CsCl isopycnic gradient centrifugation after affinity chromatography purification, still possessed residual high-density particles. In this study, we applied second CsCl isopycnic gradient centrifugation to acquire F2.2, in which low-density rAAV is no longer involved in the preparation. Thus, highly purified rAAV particles with high potency were thus acquired, indicating that the repetition of CsCl density gradient centrifugation or its effective optimization is necessary for the preparation of AAV with higher potency from a heterogeneous AAV full-particle sample that is in fact a mixture of AAV full particles with different VP stoichiometries.

Amino acid replacement at both the VP3 and VP3 clip transcription start sites in rAAV2 resulted in homogeneous rAAV capsids with high VP1 and VP2 stoichiometry. Leucine (rAAV2-M203L and rAAV2-M211L)^34^ and alanine (rAAV2-M211A) substitutions did not confirm particle formation, even though some serotypes were conserved at residues 203 and 211, although the N-terminal of VP3 homology was less (Figures 5A and S7). Homogeneous particle formation was seen with valine substitution, suggesting that a very sophisticated balance of 60-mer AAV capsid particles is being formed. The stoichiometry of VP1 + VP2 was higher for rAAV2-M203V, rAAV2-M211V, and WT rAAV in the bulk state, in descending order. The changes in stoichiometry of rAAV2-M203V and rAAV2-M211V are probably related to the translation process itself. Bosma et al. reported that different contexts of VP1 translation initiation sites alter the amount of VP3 incorporated by altering the level of VP1 translation.^24^ In such cases, a decrease in VP1 expression could result in less VP1 and VP2 incorporation and more VP3 incorporation. Meanwhile, in this study, we introduced an amino acid replacement at the start site of VP3; the decrease in VP3 translation that resulted from this could have led to the increased VP1 and VP2 incorporation for rAAV2-M203V, and similarly the lack of VP3 clip translation volume could have resulted in the slight increase in VP1 and VP2 incorporation for AAV2-M211V, based on the observation of the VP1 and VP2 stoichiometry being higher in rAAV2-M203V, rAAV2-M211V, and rAAV2-WT, in that order.

The observation of VP3 in rAAV2-M203V could be explained by the recognition of the CTG sequence by the ribosome at the original VP3 translation start position as a non-canonical start codon (Figures 3C and 3D). Once the ribosome binds to an mRNA, it moves forward until it finds the translation initiation site (ATG in the case of the canonical site) in the appropriate context and initiates protein synthesis. When weak initiation sites (e.g., ACG and CTG) are surrounded by a favorable nucleotide context, they can initiate protein synthesis in a non-canonical manner. This mechanism, called leaky ribosome scanning, has recently attracted substantial attention in the engineering of protein expression.^39^^,^^40^ In fact, a study on nucleosomes confirmed that CTG is a non-canonical translation start site in approximately 7.5% of all translation start sites in HEK293T cells,^41^ which were used for the production of rAAV in this study. As discussed in the previous study,^8^^,^^9^ Kozak sequences regulate the expression levels of VP3 and VP3 clip. If there are two possible initiation codons to be translated, the initiation codon that has A at the −3 position besides G at the +4 position (A in the initiation codon AUG is counted as +1) is preferentially used for expression.^42^^,^^43^^,^^44^ In the case of VP3 of some AAV serotypes, the first ATG at M203 has A at the −3 position and G at the +4 position (strong Kozak sequence), whereas the second ATG at M211 has C at −3 and G at +4 (weak Kozak sequence). Therefore, the population of VP3 is much larger than that of VP3 clip. In summary, this study showed experimentally that a portion of the ribosomal subunit reaches the VP3 second start codon at M211 after missing the VP2 non-canonical start codon and the VP3 start codon, resulting in VP3 clip expression, while the strong Kozak sequence at M203 results in significant VP3 translation in the WT. VP3 clip was shown experimentally to be significantly translated in the WT. The ATG to CTG mutation at position 203 could cause the low translation of VP3 of WT rAAV starting from M203 because of the lower frequency of the ribosome being trapped by the strong Kozak sequence, resulting in the translation at a non-canonical translation initiation codon. This was indicated by the deletion of the valine residue at position 203 and the acetylation of the alanine residue at the N terminus (see Table S1). On the other hand, the change from ATG to CTG at position 211 resulted in a weak initiation codon and a weak Kozak sequence that did not translate the VP3 clip, which was generated through the mechanism mediated by the non-canonical initiation codon, resulting in rAAV2-M211V for the three VPs.

The use of flow cytometry to evaluate the *in vitro* transduction efficacy showed that rAAV2-M203V and rAAV2-M211V could transduce genes into HeLa cells with higher efficiency (up to 24.7% and 21.9%, respectively) than the bulk WT rAAV2. Considering the clear relationship between the transduction efficiency and the VP1 and VP2 stoichiometry seen in F1.1 and F2.2, this result is attributed to the increased VP1 and VP2 stoichiometry in the homogeneous particles of these rAAV variants. In a previous report, mutations at the M203 and M211 positions were shown to affect particle stability and assembly.^34^ In this study, we examined the thermostability of the particles that corresponds to the structural stability of the capsid. The results indicated no significant difference in denaturation temperatures in DSF between WT rAAV2 and these rAAV2 variants. The fact that the *T*_m_ values were similar to that of WT rAAV suggests the increase in VP1 and VP2 stoichiometry, rather than the impact on the capsid structural stability, is solely responsible for the increased transduction efficacy.

Wörner et al. proposed a VP stoichiometry model based on native mass spectrometry data and parallel spectral simulations, which predicted that the compositional stoichiometry of VP subunits of AAV capsids is broadly distributed.^15^ Our CsCl isopycnic gradient ultracentrifugation succeeded in the fractionation of particles with different VP stoichiometries that constitute the WT bulk. CsCl-DG-AUC analysis also showed that the difference in the theoretical *vbar* in water between F1.1 and F2.2 based on VP stoichiometry determined by CGE with the same encapsidated ssDNA was 0.0003 cm^3^/g (F1.1: 0.6839 cm^3^/g and F2.2: 0.6837 cm^3^/g), while the difference in *vbar* in CsCl isopycnic point in the experiment was 0.0049 cm^3^/g (F1.1: 0.7396 cm^3^/g and F2.2: 0.7347 cm^3^/g). This suggests that the difference in *vbar* due to differences in VP stoichiometry was significantly enhanced in the CsCl condition. However, particle stoichiometry distributions of rAAV2-M203V and rAAV2-M211V converged to homogeneity in the CsCl DG-AUC. Consistent with this, rAAV2-M203V and rAAV2-M211V showed only one band during purification using CsCl density gradient ultracentrifugation (DG-UC). The CDMS results also indicated particle VP homogeneity of the variants, mass histograms of rAAV2-M203V and rAAV2-M211V exhibited FWHM of 0.13 MDa and 0.16 MDa, respectively, indicating a narrower mass distribution than that of rAAV2-WT (0.18 MDa) (Figure 7B). Recently, it has been shown that VP ratios depend on the production system, with VP1 expression generally being lower in baculovirus production systems.^45^^,^^46^ The VP1 and VP2 ratio has also been found to be higher in rAAV produced by HEK production systems than in that produced in baculovirus production systems.^15^ It would be reasonable to infer that the distribution of VP stoichiometry in rAAV particles was affected by the levels of VP3 and VP3 clip translation, although all rAAV2 was produced in HEK293T cells. The exact mechanism of AAV particle assembly and the reduced particle heterogeneity remains unresolved.

In this study, the VP stoichiometry was well associated with *in vitro* transduction efficiency, as confirmed by the quantitative analysis of VP stoichiometry regarding the VP1 and VP2 stoichiometry and the transduction efficiency analysis. Two regions in VP1, one of which is also present in VP2, are postulated to be responsible for the transduction efficiency of AAV. The N-terminal region of VP1, normally embedded in the capsid and exposed during intracellular transport of the vector, plays an important role in the translocation of vector to the nucleus.^10^^,^^13^^,^^20^^,^^22^^,^^23^ Phospholipase domains in this region specifically hydrolyze the 2-acyl ester (sn-2) bonds of phospholipids that form the lipid bilayer of the endosome, resulting in endosomal escape of AAV. The high level of VP1 incorporation into the capsid is expected to improve the efficiency of AAV-based DNA transport into the cell nucleus. Here, when we looked only at the stoichiometry of VP1, this did not explain the *in vitro* transduction efficacy. Therefore, we also considered the contribution of NLS, another functional region of VP1 that is also present in VP2. Upon applying this approach, we found a complete correlation between the total sum of VP1 and VP2 and the transduction efficacy. Thus, VP1 and VP2 stoichiometry is important for rAAV to achieve high transduction efficiency.

In this study, the AAV particles, originally known to be heterogeneous, differed in VP stoichiometry, and two cycles of ultracentrifugation enabled their fractionation into rAAV particles with high VP1 and VP2 stoichiometry and high potency. The important roles played by the total increase in VP1 and VP2 is associated with both possessing an NLS, which is responsible for the transportation of rAAV and a PLA2 domain, responsible for endosomal escape. This study showed the successful creation of variants with a high VP1 and VP2 stoichiometry and with particle homogeneity. These variants with high VP1 and VP2 stoichiometry are better suited to the more scalable LC-based purification. This is currently a focus of development for achieving large-scale production. In recent years, while capsid engineering for well-directed tropism has been conducted, manufacturability is critical. Therefore, variants with high potency and high manufacturability, such as those presented in this study, will become more important from a manufacturing perspective in the future.

## Materials and methods

### Cell culture

Suspended HEK293T cells were used for AAV vector production. In addition, HeLaRC32 Cells were used for *in vitro* characterization. Suspended HEK293T cells were maintained with BalanCD HEK293 (FUJIFILM Irvine Scientific, Inc., Santa Ana, CA) with 1% penicillin-streptomycin. HeLaRC32 cells were maintained in DMEM (Sigma-Aldrich, St. Louis, MO) supplemented with 10% fetal bovine serum (FBS, Hyclone [GE Healthcare Life Sciences], Madison, WI) and 1% penicillin-streptomycin (Gibco [Thermo Fisher Scientific], Grand Island, NY). Cells were grown as adherent cultures in 5% CO_2_ at 37°C.

### Construction of variant plasmid

The translational start of the VP3 and VP3 clip was modified through site-directed mutagenesis. For each variant plasmid, two complementary PCR primers, containing a missense mutation in the individual capsid protein in the start codons were used to introduce changes in the *cap* open reading frame. These plasmids were screened for restriction sites inserted by silent mutations, and the mutations were confirmed through DNA sequencing.

### rAAV preparation

All rAAV vectors were generated using the triple plasmid, co-transfection. Briefly, pAAV-Rep&Cap (serotype 2), pAd helper, and transgene (CMV-EGFP) plasmids (Vector Builder, Vector ID: VB010000-9394npt) were co-transfected into suspended HEK293T cells cultured in a bioreactor at a ratio of 1:1:1. Vectors from the transfected cells and the medium were harvested 72 h post-transfection and purified through affinity chromatography using AAVx columns (Thermo Fisher Scientific, Waltham, MA). Bulk rAAV samples were purified using affinity chromatographic purification followed by a single CsCl ultracentrifugation to separate full and empty particles. The rAAV samples purified through affinity chromatography (>2 mL) were transferred to 13.2 mL ultra-clear ultracentrifuge tubes containing 2.5 M CsCl/PBS with 0.001 w/v% poloxamer-188 solution, bringing the final volume to 12 mL. The tubes were centrifuged at 34,000 rpm in an Optima XE-90 (Beckman Coulter, Inc., Brea, CA) using a Beckman SW41Ti rotor at 20°C for 72 h. The virus band was collected with an online monitoring apparatus and dialyzed in Slide-A-Lyzer 10K (Thermo Fisher Scientific).

### Two-cycle CsCl DG-UC purification

We conducted two-cycle CsCl DG-UC to separate and fractionate high- and low-density full particles. The purified rAAV samples (>2 mL) were transferred to 13.2-mL ultra-clear ultracentrifuge tubes containing 2.5 M CsCl/PBS with 0.001 w/v% poloxamer-188 solution, bringing the final volume to 12 mL. The tubes were then centrifuged at 34,000 rpm in an Optima XE-90 (Beckman Coulter) using a Beckman SW41Ti rotor at 20°C for 72 h. The virus band containing the enriched complete particles was extracted and mixed with 2.5 M CsCl/PBS with 0.001 w/v% poloxamer-188 solution and subjected to another round of ultracentrifugation at 24,000 rpm for 72 h. The virus band was collected with an online monitoring apparatus and dialyzed in Slide-A-Lyzer 10K (Thermo Fisher Scientific).

### CGE for ssDNA

We treated 10 μL AAV solution (1.0 × 10^12^ viral genome) with DNAase and ProteaseK to extract the ssDNA from the capsid using the following protocol. To prepare the rAAV sample, 20 μL of nuclease-free water, 3 μL of 10× DNase buffer, 1.5 μL of Benzonase, and 5.5 μL of 1× PBS with 0.001% P-188 were mixed to obtain a final volume of 30 μL. Then, the sample was incubated at 37°C for 30 min. Thereafter, 30 μL of DNase-treated AAVs were transferred to a new tube, to which 10 μL of 500 mM EDTA, 55 μL of 1× PBS with 0.001% P-188, and 5 μL of Proteinase K (20 mg/mL) were added to obtain a final volume of 100 μL. Next, the mixture was incubated at 55°C for 60 min, after which the mixture was heated at 95°C for 20 min, followed by centrifugation to collect the lysate. Then, the ssDNA was purified according to the protocol mentioned in the QIAquick PCR Purification Kit (QIAGEN, Hilden, Germany) and used as the final collected sample. CGE was measured using a PA800Plus system (Sciex, Framingham, MA). The prepared samples were injected via electrokinetic injection. Detection was performed using a 488-nm laser excitation fluorescence with an emission filter of 520 nm.

### CGE for VP components

AAV samples for CGE measurement were prepared mostly in accordance with a previously reported procedure.^29^ AAV solutions with a volume of 10 μL (5.0 × 10^10^ viral genomes) were denatured and buffer-exchanged following the protocol and the final collected sample was diluted with 50 μL of deionized water for injection. CGE measurement was performed using a PA800Plus system (Sciex). Prepared samples were injected with water plug sample stacking. Detection was performed at 214 nm using a photo diode array detector.

### CDMS analysis with intact capsid

Prior to analysis by CDMS, 25 μL of each sample was directly buffer-exchanged into 200 mM aqueous ammonium acetate (Invitrogen, AM9070G) with 0.01% pluronic F-68 (ThermoFisher Scientific, 24040032) using Micro Bio-Spin P-6 gel columns (Bio-Rad, 7326221).

Mass analysis was performed using a prototype Charge Detection-Mass Spectrometer with an electrostatic linear ion trap that is based on the system built by Megadalton Solutions, which has been described previously.^47^^,^^48^^,^^49^^,^^50^ Ions were generated by positive mode nanoelectrospray ionization using a TriVersa NanoMate (Advion, Ithaca, NY), equipped with a 5-μm ID nozzle and standard A chip. The mass range of CDMS system was calibrated using L-glutamate dehydrogenase (GDH) from bovine liver (Sigma-Aldrich, G7882). To generate this calibration, the *m/z* peaks in the GDH spectra were plotted against the theoretical *m/z* values of GDH to give a correction factor that was used to accurately determine the mass and charge from the CDMS system. The NanoMate aspirated 5 μL (5.0 × 10^12^ vg/mL) of each sample to be infused into CDMS system with 1.75 kV applied to the nozzle. The spectra were collected until approximately 3,000 ions were captured within the mass range of 3–5 MDa, with a total acquisition time of approximately 10–12 min. Signal processing and data visualization were performed using prototype software developed in-house.

### LC-MS with intact VPs

rAAV samples were denatured with 10% acetic acid (FUJIFILM Wako Chemicals, Hyogo, Japan) and incubated at room temperature for 15 min. For the on-column denaturation experiment, AAV original solutions were directly used without acetic acid treatment. AAV samples were injected to Nexra HPLC (Shimadzu, Kyoto, Japan) coupled with a maXis II ETD ESI-QTOF mass spectrometer (Bruker). The separation was performed on an ACQUITY BEH C4 column (300 Å, 1.7 μm, 2.1 mm × 150 mm; Waters, Milford, MA) at a flow rate of 0.2 mL/min and temperature of 80°C. Mobile phases A and B were 0.1% difluoroacetic acid (Waters) in MS-grade water (Kanto Kagaku Co., Ltd., Tokyo, Japan) and in acetonitrile (Thermo Fisher Scientific), respectively. The pH values of solution A and a mixture of 95% solution A and 5% solution B were measured as 1.93 and 1.90, respectively. Intact VPs were eluted from a 32% B to 36% B gradient in 15 min. UV adsorption at 280 nm and intrinsic fluorescence of aromatic amino acids with the excitation wavelength set at 280 nm and the emission wavelength set at 350 nm were used for detection of the LC chromatogram. Deconvolution analysis was performed in the mass range of 40,000–90,000 Da to determine the mass values of VP components in the eluted peaks. Visualization and processing of mass spectra were performed using Bruker Compass Data Analysis software ver. 5.1 (Bruker) and the maximum entropy was used for deconvolution.

### CsCl-DG-AUC

Samples were dissolved in a CsCl solution of the set prepared concentration in PBS with 0.001 w/v% poloxamer-188 as the solvent. Each rAAV stock was diluted to a final absorbance at a 1-cm path length of approximately 0.1 at 230 nm. A total of 390 μL of the sample was loaded into the sample sector equipped with sapphire windows and a 12-mm double-sector charcoal-filled epon centerpiece (Beckman Coulter, USA). Further, 400 μL of a corresponding CsCl solvent was loaded into each reference sector.

Data were collected at 20°C using Optima AUC (Beckman Coulter) at 42,000 rpm with a UV-Visible absorption detection system every hour for up to 72 h with a radial increment of 10 μm.

### Determination of *T*_m_

Structural stabilities of rAAV capsid were assessed by DSF using Uncle (Unchained Labs, Pleasanton, CA). Nine microliters of the rAAV2 samples were added to special measurement wells called Uni. Intrinsic fluorescence of aromatic amino acids (mainly from Trp^51^) excited by a 266-nm laser was measured as the temperature was increased at a rate of 1 °C/min. A redshift, involving the wavelength shifting to a greater value because of an environmental change of Trp residues from hydrophobic surroundings to hydrophilic ones during protein denaturation, was detected by plotting temperature on the y axis and barycentric mean (BCM) of the fluorescence from 300 to 430 nm on the x axis. The point at which the integral value of BCM peaked was defined as *T*_m_.

### BS-AUC

BS-AUC experiments and analysis were performed in accordance with our previously reported method.^31^ Briefly, AAV stocks were diluted to a final absorbance at a 1-cm path length of 0.25 at 260 nm. Then, 15 μL of the AAV vector solution and solvent were loaded into a sample or reference reservoir well with a 12-mm band-forming centerpiece (Spin Analytical, Berwick, ME) equipped with sapphire windows. A volume of 240 μL or 250 μL of PBS/D_2_O with 0.001% w/v poloxamer-188 was loaded into the sample or reference sector, respectively. Data were collected at 20°C using the Optima AUC (Beckman Coulter) at 20,000 rpm with a UV detection system. Data were collected immediately with a radial increment of 10 μm. The BS-AUC sedimentation data were analyzed using the analytical zone centrifugation c(s) model of the program SEDFIT (version 16.2b), where the lamella width, frictional ratio, meniscus, time-invariant noise, and radial-invariant noise were fitted using a regularization level of 0.68. The s-value range of 0–175 S was evaluated with a resolution of 350, and the buffer density and viscosity of PBS/H_2_^18^O were calculated using the program SEDNTERP. The figures of the c(s) distribution were generated using the program GUSSI (version 1.3.2). The s-value was described as *sw* (s-value as an apparent value under the experimental conditions).

### *In vitro* transduction efficacy assay

HeLaRC32 expressing the *Rep* and *Cap* genes for rAAV replication. After culture, HeLaRC cells were seeded in 24-well culture plates (Corning Inc., NY) at 5 × 10^4^ cells/well. The next day, cells were observed using a microscope to check that they were uniformly attached to the surface in all plates. Cells were infected with the AAV vectors at MOI 1 × 10^2^, 2.5 × 10^3^, 1 × 10^3^, 2.5 × 10^3^, and 5 × 10^3^ in triplicate. The amounts of rAAV solutions were determined according to the calculated cell counts in three randomly selected wells. After 5 h, complete medium (DMEM, 10% FBS, 1% P/S) was added for further culture. At 72 h post-infection, cells were washed with DPBS (Wako, Osaka, Japan) and detached using TrypLE Select Enzyme (1X), with no phenol red (Thermo Fisher Scientific). Trypsin digestion was terminated by adding complete medium. Analysis of GFP expression was performed in a CytoFlex II Flow Cytometer (Beckman Coulter) using a fluorescein isothiocyanate (excitation: 498 nm, emission: 522 nm) channel with a threshold of 10,000 being used to identify GFP-positive cells.

### Real-time qPCR

rAAV vectors were quantified by qPCR in QuantStudio 3 Real-Time PCR System (Thermo Fisher Scientific) using AAVpro Titration Kit (for Real-Time PCR) Ver. 2 (Takara, Tokyo, Japan). Almost all steps were processed according to the manual. Briefly, DNase I treatment was performed to digest DNA outside the capsid, following heat inactivation of enzyme and capsid denaturation. The extracted viral genome was diluted and mixed with solution containing TB Green intercalating dye. A dilution series of the positive control (2 × 10^7^ to 2 × 10^2^ copies/μL) was measured to generate a standard curve. Two minutes of initial denaturation at 95°C was followed by 35 cycles of denaturation at 95°C for 5 s and annealing at 60°C for 30 s, with final melting curve analysis. Viral titers were calculated in technical duplicates as genomic particles per milliliter.

### Productivity assay

WT rAAV and rAAV variants were produced in 20-mL flasks (Corning, Corning, NY, USA) using suspended HEK293T cells (three flasks per rAAV type). The cells were subjected to triple transfection according to established protocols. Then, the cells were allowed to grow for 24, 48, and 72 h in an incubator. At each time point, the cells were collected through centrifugation and the cell pellet was resuspended in AAV-MAX lysis buffer to obtain a lysate containing rAAV particles. Real-time qPCR was used to quantify the rAAV titer in each lysate. Finally, the productivity of WT and rAAV variants was compared based on their respective titers.

### Image processing of graphical abstract figure

The rAAV2 structure, PDB ID: 1LP3 (60-mer), was used as template. The visualization of X-ray crystallography maps and image making was performed using 3D Protein Imaging.^52^

## Data and code availability

Data will be made available on request.

## Supplemental information

Document S1. Figures S1–S7, Tables S1, S2, Equations S1 and S2

Document S2. Article plus supplemental information

## References

[1] Blair H.A. (2022). Valoctocogene Roxaparvovec: First Approval. Drugs.

[2] Deng C., Zhao P.Y., Branham K., Schlegel D., Fahim A.T., Jayasundera T.K., Khan N., Besirli C.G. (2022). Real-world outcomes of voretigene neparvovec treatment in pediatric patients with RPE65-associated Leber congenital amaurosis. Graefes Arch. Clin. Exp. Ophthalmol..

[3] Keam S.J. (2022). Eladocagene Exuparvovec: First Approval. Drugs.

[4] Keeler A.M., Flotte T.R. (2019). Recombinant Adeno-Associated Virus Gene Therapy in Light of Luxturna (and Zolgensma and Glybera): Where Are We, and How Did We Get Here?. Annu. Rev. Virol..

[5] Schwartz M., Likhite S., Meyer K. (2021). Onasemnogene abeparvovec-xioi: a gene replacement strategy for the treatment of infants diagnosed with spinal muscular atrophy. Drugs Today.

[6] Buller R.M., Rose J.A. (1978). Characterization of adenovirus-associated virus-induced polypeptides in KB cells. J. Virol..

[7] Johnson F.B., Ozer H.L., Hoggan M.D. (1971). Structural proteins of adenovirus-associated virus type 3. J. Virol..

[8] Jin X., Liu L., Nass S., O'Riordan C., Pastor E., Zhang X.K. (2017). Direct Liquid Chromatography/Mass Spectrometry Analysis for Complete Characterization of Recombinant Adeno-Associated Virus Capsid Proteins. Hum. Gene Ther. Methods.

[9] Oyama H., Ishii K., Maruno T., Torisu T., Uchiyama S. (2021). Characterization of Adeno-Associated Virus Capsid Proteins with Two Types of VP3-Related Components by Capillary Gel Electrophoresis and Mass Spectrometry. Hum. Gene Ther..

[10] Sonntag F., Bleker S., Leuchs B., Fischer R., Kleinschmidt J.A. (2006). Adeno-associated virus type 2 capsids with externalized VP1/VP2 trafficking domains are generated prior to passage through the cytoplasm and are maintained until uncoating occurs in the nucleus. J. Virol..

[11] Venkatakrishnan B., Yarbrough J., Domsic J., Bennett A., Bothner B., Kozyreva O.G., Samulski R.J., Muzyczka N., McKenna R., Agbandje-McKenna M. (2013). Structure and dynamics of adeno-associated virus serotype 1 VP1-unique N-terminal domain and its role in capsid trafficking. J. Virol..

[12] Bleker S., Sonntag F., Kleinschmidt J.A. (2005). Mutational analysis of narrow pores at the fivefold symmetry axes of adeno-associated virus type 2 capsids reveals a dual role in genome packaging and activation of phospholipase A2 activity. J. Virol..

[13] Girod A., Wobus C.E., Zádori Z., Ried M., Leike K., Tijssen P., Kleinschmidt J.A., Hallek M. (2002). The VP1 capsid protein of adeno-associated virus type 2 is carrying a phospholipase A2 domain required for virus infectivity. J. Gen. Virol..

[14] Pierson E.E., Keifer D.Z., Asokan A., Jarrold M.F. (2016). Resolving Adeno-Associated Viral Particle Diversity With Charge Detection Mass Spectrometry. Anal. Chem..

[15] Wörner T.P., Bennett A., Habka S., Snijder J., Friese O., Powers T., Agbandje-McKenna M., Heck A.J.R. (2021). Adeno-associated virus capsid assembly is divergent and stochastic. Nat. Commun..

[16] Wang Q., Firrman J., Wu Z., Pokiniewski K.A., Valencia C.A., Wang H., Wei H., Zhuang Z., Liu L., Wunder S.L. (2016). High-Density Recombinant Adeno-Associated Viral Particles are Competent Vectors for In Vivo Transduction. Hum. Gene Ther..

[17] Lipps B.V., Mayor H.D. (1982). Characterization of heavy particles of adeno-associated virus type 1. J. Gen. Virol..

[18] Torikai K., Ito M., Jordan L.E., Mayor H.D. (1970). Properties of light particles produced during growth of Type 4 adeno-associated satellite virus. J. Virol..

[19] de la Maza L.M., Carter B.J. (1980). Heavy and light particles of adeno-associated virus. J. Virol..

[20] Grieger J.C., Johnson J.S., Gurda-Whitaker B., Agbandje-McKenna M., Samulski R.J. (2007). Surface-exposed adeno-associated virus Vp1-NLS capsid fusion protein rescues infectivity of noninfectious wild-type Vp2/Vp3 and Vp3-only capsids but not that of fivefold pore mutant virions. J. Virol..

[21] Grieger J.C., Snowdy S., Samulski R.J. (2006). Separate basic region motifs within the adeno-associated virus capsid proteins are essential for infectivity and assembly. J. Virol..

[22] Xiao P.J., Samulski R.J. (2012). Cytoplasmic trafficking, endosomal escape, and perinuclear accumulation of adeno-associated virus type 2 particles are facilitated by microtubule network. J. Virol..

[23] Popa-Wagner R., Porwal M., Kann M., Reuss M., Weimer M., Florin L., Kleinschmidt J.A. (2012). Impact of VP1-specific protein sequence motifs on adeno-associated virus type 2 intracellular trafficking and nuclear entry. J. Virol..

[24] Bosma B., du Plessis F., Ehlert E., Nijmeijer B., de Haan M., Petry H., Lubelski J. (2018). Optimization of viral protein ratios for production of rAAV serotype 5 in the baculovirus system. Gene Ther..

[25] Mietzsch M., Grasse S., Zurawski C., Weger S., Bennett A., Agbandje-McKenna M., Muzyczka N., Zolotukhin S., Heilbronn R. (2014). OneBac: platform for scalable and high-titer production of adeno-associated virus serotype 1-12 vectors for gene therapy. Hum. Gene Ther..

[26] Srivastava A., Mallela K.M.G., Deorkar N., Brophy G. (2021). Manufacturing Challenges and Rational Formulation Development for AAV Viral Vectors. J. Pharmaceut. Sci..

[27] Florea M., Nicolaou F., Pacouret S., Zinn E.M., Sanmiguel J., Andres-Mateos E., Unzu C., Wagers A.J., Vandenberghe L.H. (2023). High-efficiency purification of divergent AAV serotypes using AAVX affinity chromatography. Mol. Ther. Methods Clin. Dev..

[28] Lechner A., Giorgetti J., Gahoual R., Beck A., Leize-Wagner E., François Y.N. (2019). Insights from capillary electrophoresis approaches for characterization of monoclonal antibodies and antibody drug conjugates in the period 2016-2018. J. Chromatogr., B: Anal. Technol. Biomed. Life Sci..

[29] Zhang C.X., Meagher M.M. (2019). Highly Sensitive SDS Capillary Gel Electrophoresis with Sample Stacking Requiring Only Nanograms of Adeno-Associated Virus Capsid Proteins. Methods Mol. Biol..

[30] Kuipers B.J.H., Gruppen H. (2007). Prediction of molar extinction coefficients of proteins and peptides using UV absorption of the constituent amino acids at 214 nm to enable quantitative reverse phase high-performance liquid chromatography-mass spectrometry analysis. J. Agric. Food Chem..

[31] Maruno T., Ishii K., Torisu T., Uchiyama S. (2023). Size Distribution Analysis of the Adeno-Associated Virus Vector by the c(s) Analysis of Band Sedimentation Analytical Ultracentrifugation with Multiwavelength Detection. J. Pharmaceut. Sci..

[32] Jarrold M.F. (2022). Applications of Charge Detection Mass Spectrometry in Molecular Biology and Biotechnology. Chem. Rev..

[33] Barnes L.F., Draper B.E., Jarrold M.F. (2022). Analysis of thermally driven structural changes, genome release, disassembly, and aggregation of recombinant AAV by CDMS. Mol. Ther. Methods Clin. Dev..

[34] Warrington K.H., Gorbatyuk O.S., Harrison J.K., Opie S.R., Zolotukhin S., Muzyczka N. (2004). Adeno-associated virus type 2 VP2 capsid protein is nonessential and can tolerate large peptide insertions at its N terminus. J. Virol..

[35] de la Maza L.M., Carter B.J. (1980). Molecular Structureof Adeno-associated Virus Variant DNA. J. Biol. Chem..

[36] Pronovost A.D., Yates V.J., Fry D.E. (1979). Inhibition and enhancement of avian adenovirus plaque production by heavy and light avian adenovirusassociated viral particles. Am. J. Vet. Res..

[37] Paradiso P.R. (1981). Infectious process of the parvovirus H-1: correlation of protein content, particle density, and viral infectivity. J. Virol..

[38] Clinton G.M., Hayashi M. (1976). The parvovirus MVM: a comparison of heavy and light particle infectivity and their density conversion in vitro. Virology.

[39] Ferreira J.P., Noderer W.L., Diaz de Arce A.J., Wang C.L. (2014). Engineering ribosomal leaky scanning and upstream open reading frames for precise control of protein translation. Bioengineered.

[40] Hinnebusch A.G. (2011). Molecular mechanism of scanning and start codon selection in eukaryotes. Microbiol. Mol. Biol. Rev..

[41] Ichihara K., Matsumoto A., Nishida H., Kito Y., Shimizu H., Shichino Y., Iwasaki S., Imami K., Ishihama Y., Nakayama K.I. (2021). Combinatorial analysis of translation dynamics reveals eIF2 dependence of translation initiation at near-cognate codons. Nucleic Acids Res..

[42] Kozak M. (1999). Initiation of translation in prokaryotes and eukaryotes. Gene.

[43] Becerra S.P., Rose J.A., Hardy M., Baroudy B.M., Anderson C.W. (1985). Direct mapping of adeno-associated virus capsid proteins B and C: a possible ACG initiation codon. Proc. Natl. Acad. Sci. USA.

[44] Firth A.E., Brierley I. (2012). Non-canonical translation in RNA viruses. J. Gen. Virol..

[45] Kohlbrenner E., Aslanidi G., Nash K., Shklyaev S., Campbell-Thompson M., Byrne B.J., Snyder R.O., Muzyczka N., Warrington K.H., Zolotukhin S. (2005). Successful production of pseudotyped rAAV vectors using a modified baculovirus expression system. Mol. Ther..

[46] Mietzsch M., Casteleyn V., Weger S., Zolotukhin S., Heilbronn R. (2015). OneBac 2.0: Sf9 Cell Lines for Production of AAV5 Vectors with Enhanced Infectivity and Minimal Encapsidation of Foreign DNA. Hum. Gene Ther..

[47] Draper B.E., Jarrold M.F. (2019). Real-Time Analysis and Signal Optimization for Charge Detection Mass Spectrometry. J. Am. Soc. Mass Spectrom..

[48] Todd A.R., Jarrold M.F. (2020). Dynamic Calibration Enables High-Accuracy Charge Measurements on Individual Ions for Charge Detection Mass Spectrometry. J. Am. Soc. Mass Spectrom..

[49] Todd A.R., Alexander A.W., Jarrold M.F. (2020). Implementation of a Charge-Sensitive Amplifier without a Feedback Resistor for Charge Detection Mass Spectrometry Reduces Noise and Enables Detection of Individual Ions Carrying a Single Charge. J. Am. Soc. Mass Spectrom..

[50] Hogan J.A., Jarrold M.F. (2018). Optimized Electrostatic Linear Ion Trap for Charge Detection Mass Spectrometry. J. Am. Soc. Mass Spectrom..

[51] Ghisaidoobe A.B.T., Chung S.J. (2014). Intrinsic tryptophan fluorescence in the detection and analysis of proteins: a focus on Forster resonance energy transfer techniques. Int. J. Mol. Sci..

[52] Tomasello G., Armenia I., Molla G. (2020). The Protein Imager: a full-featured online molecular viewer interface with server-side HQ-rendering capabilities. Bioinformatics.

