# Supplementary material for: Enhancement of recombinant adeno-associated virus activity by improved stoichiometry and homogeneity of capsid protein assembly
Source: Mol Ther Methods Clin Dev. 2023 Oct 24;31:101142. doi: 10.1016/j.omtm.2023.101142 (PMC10663676; doi:10.1016/j.omtm.2023.101142)
Supplement: Document S1. Figures S1–S7, Tables S1, S2, Equations S1 and S2 [file mmc1.pdf]

OMTM, Volume *31*

Supplemental information

Enhancement of recombinant adeno-associated virus activity by improved stoichiometry and homogeneity of capsid protein assembly

Takayuki Onishi, Michika Nonaka, Takahiro Maruno, Yuki Yamaguchi, Mitsuko Fukuhara, Tetsuo Torisu, Masaharu Maeda, Susan Abbatiello, Anisha Haris, Keith Richardson, Kevin Giles, Steve Preece, Noriko Yamano-Adachi, Takeshi Omasa, and Susumu Uchiyama

Molecular weight (Mw) estimation

The theoretical Mw values of AAV2-Empty were determined from the amino acid composition of capsid protein determined by CGE measurement using SEDNTERP. Furthermore, the theoretical Mw values of ssDNA were calculated from the DNA compositions.

In BS-AUC experiment, the Mw values of AAV2-Empty and AAV2-Full were calculated using the Svedberg equation:

$$\frac{s}{D} = \frac{M(1 - vbar\rho)}{RT} \quad \text{(Equation S1)}$$

where s is the sedimentation coefficient, D is the diffusion coefficient, M is the Mw, $vbar$ is the particle partial-specific volume, ρ is the solvent density, R is the gas constant, and T is the absolute temperature.

Partial specific volume ($vbar$) calculation

The $vbar$ values of AAV-Empty were calculated from the amino acid composition of capsid protein determined by CGE measurement.

The $vbar$ values of F1.1 and F2.2 in water can be theoretically calculated using the following equation:

$$vbar_{AAV-FP} = \frac{vbar_{AAV-Empty} \times Mw_{AAV-Empty} + vbar_{ssDNA} \times Mw_{ssDNA}}{Mw_{AAV-Empty} + Mw_{ssDNA}} \quad \text{(Equation S2)}$$

where $vbar_{AAV-F1.1\ or\ F2.2}$, $vbar_{AAV-Empty}$, and $vbar_{ssDNA}$ are the partial-specific volume of AAV-F1.1 or F2.2, AAV-Empty, and ssDNA, respectively. 0.52 $cm^3\ g^{-1}$ was used as $vbar_{ssDNA}$ based on the previous study.[5] $Mw_{AAV-F1.1\ or\ F2.2}$, $Mw_{AAV-Empty}$, and Mw_{ssDNA} are the Mw of AAV-FP1.1 or AAV-FP2.2, AAV-EP, and ssDNA, respectively.
, using partial specific volumes of 0.6840 cm^3/g and 0.6837 cm^3/g (Equation S2), respectively, and a frictional ratio (f/f_0) of 1.46 for both particles.

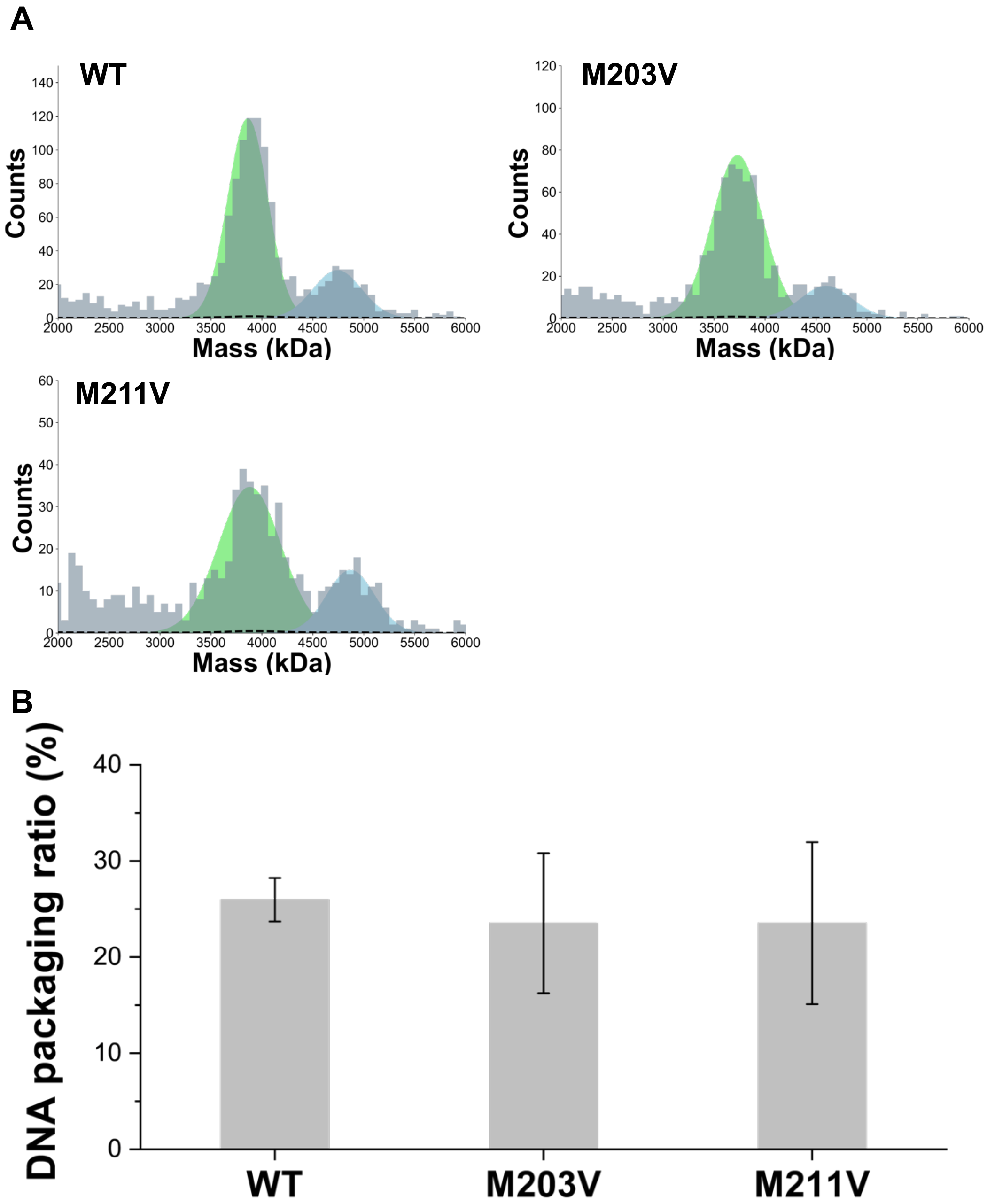

Figure S1. DNA packaging ratio of rAAV-WT, -M203V and -M211V evaluated by mass photometry (MP).

DNA packaging ratio before centrifugal purification were measured by MP. MP experiments were conducted using the TwoMP instrument (Refeyn, Oxford, UK).

A) One of the representative mass distributions of rAAV-WT, -M203V and -M211V. Show Gaussian fitting for empty particle in green for full particle in blue.

B) DNA packaging ratio of rAAV-WT, -M203V and -M211V.

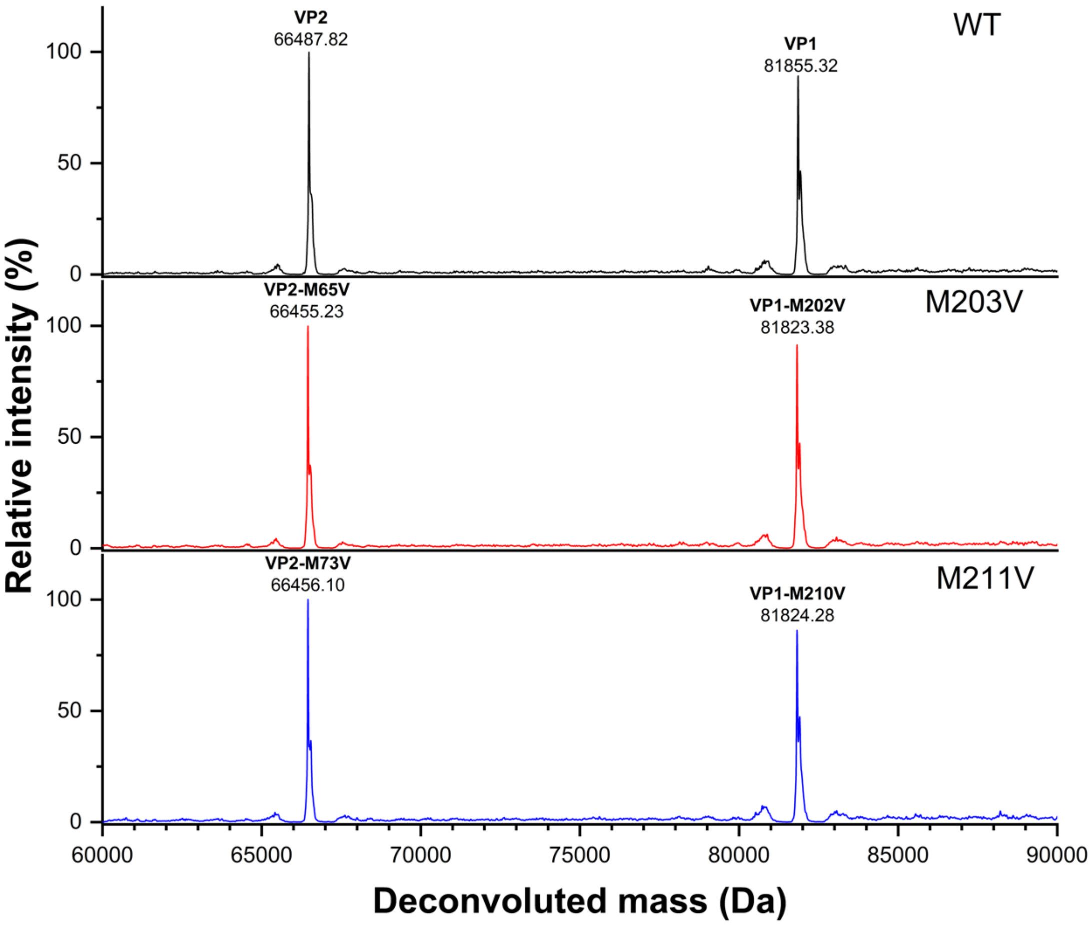

Figure S2. Deconvoluted mass values of WT, M203V, and M211V for VP1 and VP2 by liquid chromatography-mass spectrometry (LC-MS).

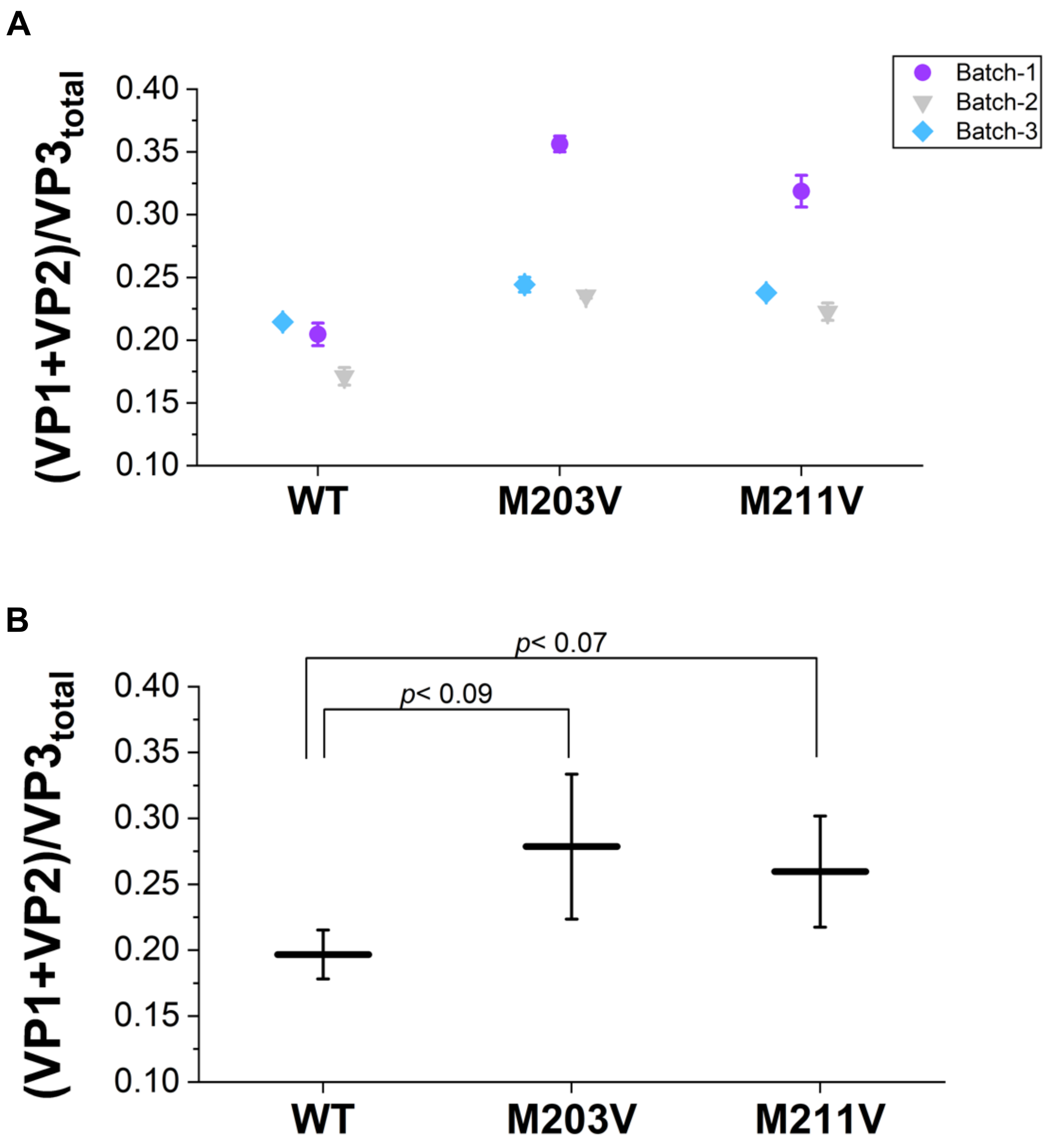

Figure S3. Variability of VP stoichiometry of rAAV2-WT, -M203V, -M211V.

A) Variability of VP stoichiometry for rAAV2-WT, -M203V, -M211V in the three different batches.

B) Means and standard deviations in the three different batches.

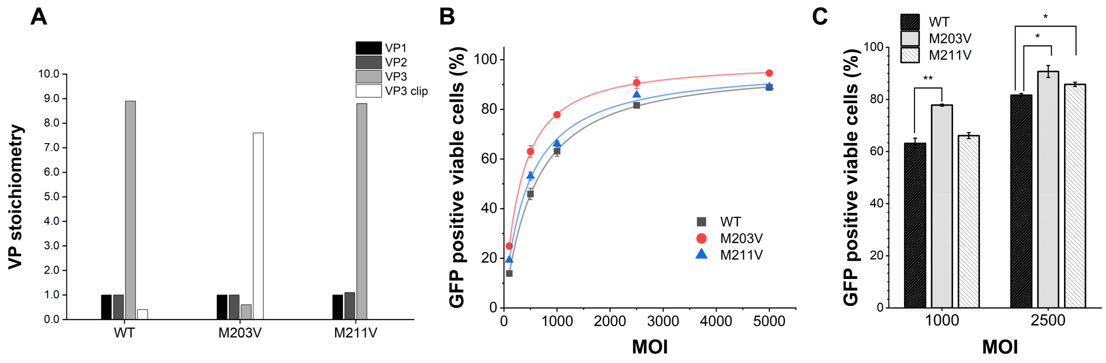

Figure S4. Characterization of rAAV2-WT, -M203V and -M211V on differential transgene.

rAAV2-M203V and -M211V with the transgene changed to CMV-zsGreen1 were prepared and evaluated VP stoichiometry and *In vitro* transduction efficacy.

A) VP stoichiometry of WT, M203V and M211V.

B) GFP positive viable cells were measured by fluor cytometer at five points of MOI (MOI 1x10^2, 5x10^2, 10x10^2, 25x10^2 and 50x10^2).

C) *In vitro* transduction efficacy of selected MOI 10x10^2 and 25x10^2.

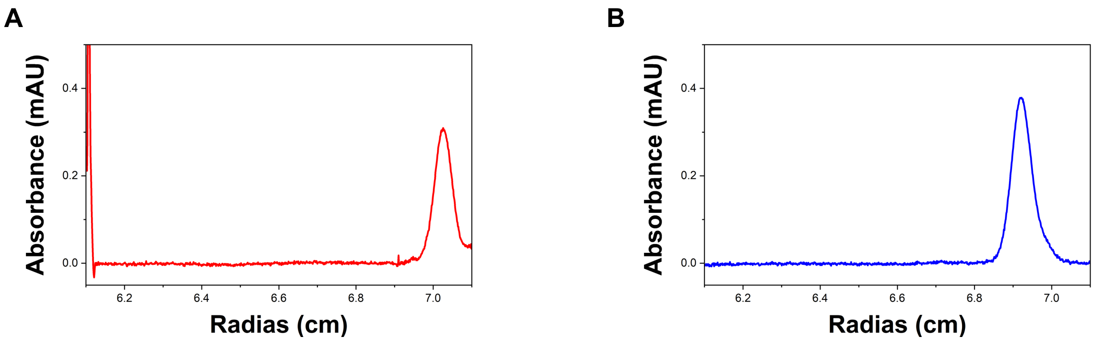

Figure S5. CsCl-density gradient analytical ultracentrifugation (CsCl-DG-AUC) profile of rAAV2-M203V and -M211V on differential transgene.

A-B were CsCl-DG-AUC profile of rAAV2 variants packaged zsGreen1 gene downstream of cytomegalovirus promoter (CMV-zsGreen1), and the single distribution was observed in both variants.

A) CsCl-DG-AUC profile of rAAV2-CMV-zsGreen1-M203V.

B) CsCl-DG-AUC profile of rAAV2-CMV-zsGreen1-M211V.

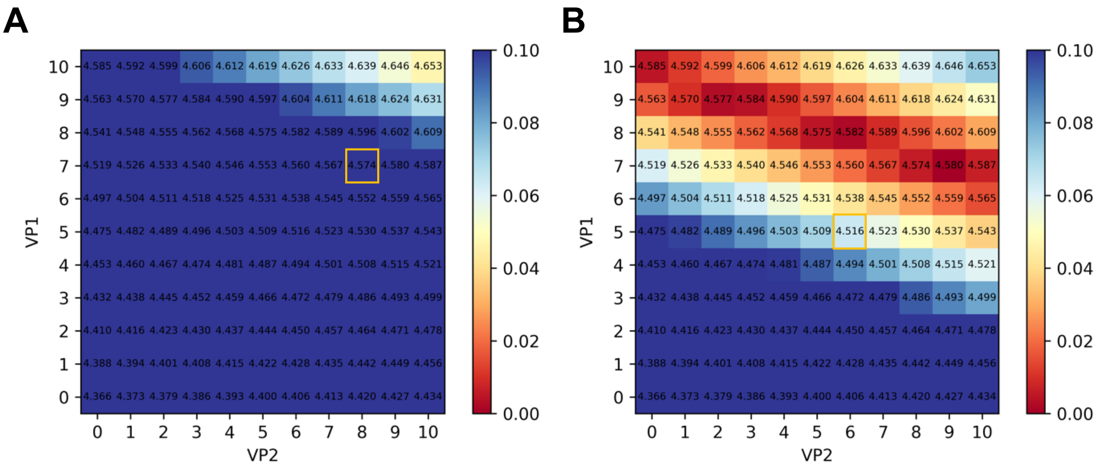

Figure S6. Estimation of VP combination in 60-mer based on particle mass by CD-MS.

Heat maps of VP matrix-mass value were created to assess the correspondence of VP1 and VP2 stoichiometry between theoretical mass values and measured mass values for particles from CD-MS analysis. Calculated mass value based on CGE experiments were also indicated.

The theoretical mass values of the corresponding VP combination were indicated in each cell, and the color chart indicates the difference from the CD-MS mass value. The mass values of all VP combinations were calculated as a 60-mer and all components except VP1 and VP2 were calculated as VP3. Yellow squares indicate mass value using VP1 and VP2 stoichiometry obtained from CGE measurement.

A) Heat map of mass difference between CD-MS mass value and each VP combination for F1.1 particles.

B) Heat map of mass difference between CD-MS mass value and each VP combination for F2.2 particles.

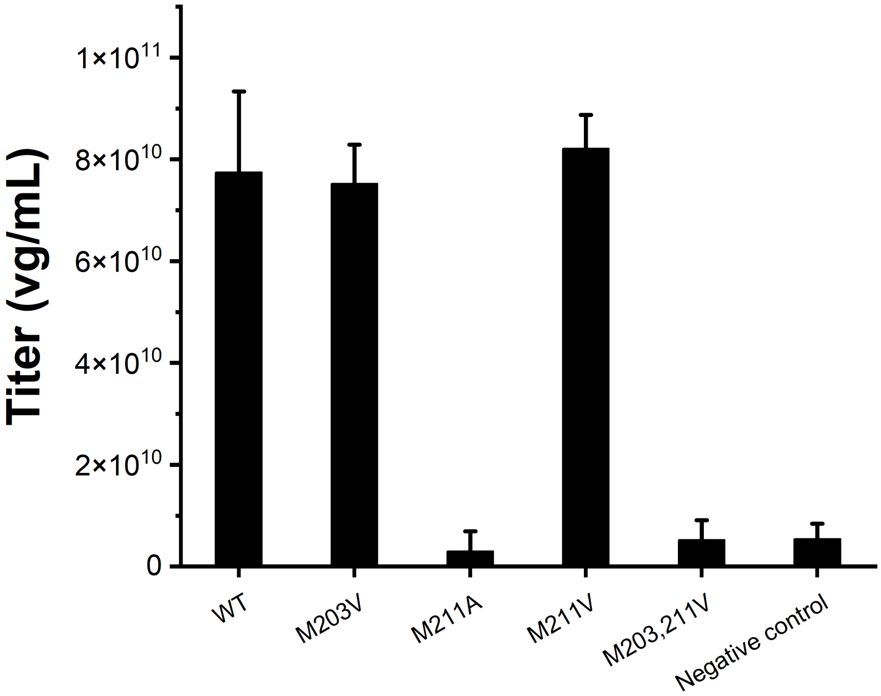

Figure S7. **Particle assembly assessment.**

To evaluate rAAV particle formation, cell lysates at 72 hours post-transfection were assayed by qPCR. Cell lysates transfected with plasmids except for the pHelper plasmid were used as negative controls, considering the possibility of insufficient digestion with Benzonase prior to qPCR measurement. Particle formation was considered to have failed in cases of titers comparable to those of the negative control.

Table S1. Comparison of theoretical and measured molecular mass.

	VP ratio from CGE (VP1:VP2:VP3:VP3clip)	Theoritical mass from CGE result* (MDa)	Molecular mass from CDMS (MDa)	Molecular weight from BS-AUC (MDa)
**AAV**	1.0 : 1.0 : 10.0 :-	4.54	-	-
**F1.1**	1.0 : 1.3 : 6.9 : 0.4	4.56	4.70	5.24
**F2.2**	1.0 : 1.4 : 8.6 : 0.4	4.53	4.58	5.11
**rAAV2-WT**	1.0 : 1.3 : 10.9 : 0.4	4.54	4.75	-
**rAAV2-M203V**	1.0 : 1.8 : 0.5 : 7.5	4.56	4.73	-
**rAAV2-M211V**	1.0 : 1.9 : 9.1 : 0	4.57	4.71	-

*Total VP number was assumed to be 60 in a single AAV particle.

*Molecular weights used for the calculation of theoretical mass value from amino acid sequence: VP1: 81855.24, VP2: 66488.20, VP3: 59974.02, $VP3_{clip}$: 59301.27 for wild-type, VP1: 81823.1875, VP2: 66456.14, VP3: 59974.02, $VP3_{clip}$: 59301.27 for rAAV2-M203V, VP1: 81823.1875, VP2: 66456.14, VP3: 59941.96 for rAAV2-M211V and the calculation of theoretical mass value from nucleic acid sequence: ITR-CMV-EGFP-ITR (2,521 bp): 777502.35.

Table S2. Estimated amino acid sequence and mass accuracy by LC-MS measurements.

	Estimated amino acid sequence	Observed mass (Da)	Theoretical mass (Da)	Mass accuracy (ppm)
**AAV2-WT**	VP1 (A2(Ac)-L735)	81855.32	81855.24	0.95
	VP2 (A139-L735)	66487.82	66488.20	5.76
	VP3 (A204(Ac)-L735)	59973.14	59974.02	14.73
	VP3 clip (A212(Ac)-L735)	59299.79	59301.27	24.95
**AAV2-M203V**	VP1-M202V (A2(Ac)-M203V-L735)	81823.38	81823.19	2.35
	VP2-M65V (A139-M203V-L735)	66455.23	66456.14	13.70
	VP3 (A204(Ac)-L735)	59972.82	59974.02	20.07
	VP3 clip (A212(Ac)-L735)	59300.22	59301.27	17.70
**AAV2-M211V**	VP1-M210V (A2(Ac)-M211V-L735)	81824.28	81823.19	13.35
	VP2-M73V (A139-M211V-L735)	66456.10	66456.14	0.61
	VP3-M8V (A204(Ac)-M211V-L735)	59941.72	59941.96	4.08
	VP3 clip (A212(Ac)-L735)	ND	-	-
